# Transport and Reaction of Electrons and Molecules in Solid Electrolyte Interphases formed at Different Electrode Potentials: A Combined Experimental and Modeling Approach

**DOI:** 10.1002/cssc.202402468

**Published:** 2025-04-02

**Authors:** Falk Thorsten Krauss, Annalena Duncker, Bernhard Roling

**Affiliations:** ^1^ Department of Chemistry Philipps-Universität Marburg Hans-Meerwein-Straße 4 35032 Marburg Germany

**Keywords:** electrochemistry, lithium-ion batteries, solid electrolyte interphase, generator-collector experiments, transport and reaction mechanisms

## Abstract

Good passivation properties of the solid electrolyte interphase (SEI) on the graphite‐based negative electrode are essential for a long cycle life of lithium‐ion batteries. Nevertheless, the underlying electron and molecule transport mechanisms inside the SEI are poorly understood. Here, we elucidate transport and reaction in model‐type SEIs formed at different electrode potentials by combining generator‐collector experiments and electrochemical impedance spectroscopy with a diffusion‐reaction modeling approach. In the generator‐collector experiments, we use a four‐electrode‐based setup to compare the electrolyte reduction current density with a redox molecule (ferrocenium Fc^+^) reduction current density at an SEI‐covered glassy carbon electrode. We find that the current density ratio depends on the SEI formation potential as well as on the formation time. The experimental results are compared to the prediction of a transport and reaction model, which accounts for reduction reactions inside the SEI as well as in the double layer at the SEI | bulk electrolyte interface. This model predicts four distinct diffusion and reaction regimes depending on the rate constant for the molecule‐electron reaction. Using this combined approach, we obtain good estimates for the transport coefficients of electrons and molecules inside the SEI.

## Graphical abstract

Transport and reaction mechanisms in model‐type SEIs are elucidated by a combined experimental and modeling approach. Current densities due to SEI formation and due to redox molecule reduction are measured simultaneously. A comparison of the experimental current density ratio with the predictions of a transport and reaction model yields good estimates for the transport coefficients of electrons and molecules.

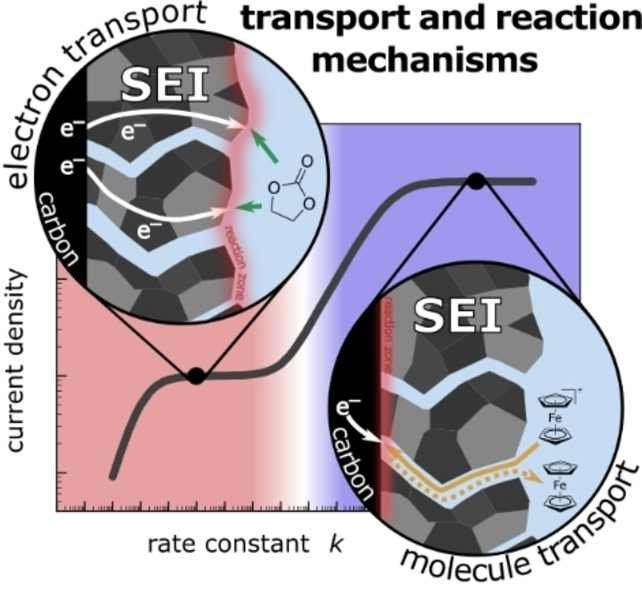

## Introduction

Lithium‐ion batteries are the state‐of‐art energy storage technology for mobile applications. In these batteries, the negative graphite‐based electrode operates outside the electrochemical stability window of the liquid electrolyte.[^1^, ^2^] During the first charging, the electrolyte is decomposed, and the reaction products form a thin passivation layer on the surface of the graphite particles, the so‐called solid electrolyte interphase (SEI).[^3^, ^4^, ^5^, ^6^, ^7^, ^8^, ^9^] A stable SEI with low ionic resistance is crucial for lithium‐ion batteries with high power density and long cycle life.^[10]^

The SEI exhibits typically a thickness in the range of some 10 nm and is a multi‐compound and multi‐phase system. The dense inner layer close to the graphite surface consists mainly of inorganic compounds, such as LiF and Li_2_CO_3_, arranged in a mosaic‐like fashion.[^6^, ^9^, ^11^] The porous outer layer of the SEI comprises mainly organic compounds, like lithium ethylene dicarbonate, semicarbonates and polycarbonates.[^9^, ^12^, ^13^]

Despite this knowledge about structure and chemical composition, the understanding of transport and reaction processes in the SEI and of the resulting passivation properties is still limited. A number of experimental publications report a parabolic law for the long‐time growth of the SEI, i. e., the SEI thickness $d_{SEI}$ increases with $\sqrt{t}$ ,[^14^, ^15^, ^16^, ^17^, ^18^] giving indication for a transported‐limited growth mechanism. Accordingly, solvent transport‐limited growth[^15^, ^19^, ^20^, ^21^, ^22^, ^23^, ^24^, ^25^, ^26^] as well as electron transport‐limited growth mechanisms[^19^, ^27^, ^28^, ^29^, ^30^, ^31^] were suggested. Recently, it has been shown that an electron transport‐limited growth mechanism provides an explanation for experimental results on calendar ageing as well as on cycling ageing of batteries.[^19^, ^31^, ^32^] This mechanism is based on an electrode potential‐dependent electron concentration at the electrode | SEI interface, leading to a diffusive electron transport across the SEI and to a reduction of solvent molecules at the SEI | bulk electrolyte interface.

Although the SEI passivates well with regard to electrolyte reduction, it has been observed that reduction of redox shuttle additives at the SEI‐covered negative electrode is sufficiently fast for establishing an internal overcharge protection in lithium‐ion batteries.[^33^, ^34^, ^35^] These distinct passivation properties of the SEI with regard to electrolyte reduction and with regard to redox molecule reduction are not yet understood and have not yet been described in transport and passivation models.

In a resent experimental study, Krauss et al.^[36]^ formed model‐type SEIs on glassy carbon electrodes at a potential of 0.8 V vs. Li^+^/Li and measured simultaneously the reduction of the electrolyte and the reduction of redox molecules by means of a generator‐collector setup. The study confirmed distinct passivation properties of the SEI with regard to electrolyte reduction and with regard to redox molecule reduction. In particular, at short SEI formation times in the range of a few hours, the redox molecule reduction current was much higher than the electrolyte reduction current. This discrepancy was explained by distinct transport and reaction mechanisms of the molecules caused by different rate constants for the reaction of molecules with electrons. The experimental results gave strong indication for a transition of the redox molecule reduction from the electrode | SEI interface on short time scales to the SEI | bulk electrolyte interface on long time scales.

Here we extend this experimental approach to model‐type SEIs formed at different electrode potentials. Using a combination of potentiostatic SEI formation, generator‐collector experiments and electrochemical impedance spectroscopy, we measure the time‐dependent reduction currents of electrolyte molecules and of redox molecules, and we obtain information on the time‐dependent SEI thickness. For the interpretation of the results, we expand a transport and reaction model, which was developed by Savéant and coworkers for redox polymer and electrocatalytic films,[^37^, ^38^, ^39^, ^40^, ^41^, ^42^] by additionally taking into account reactions within the double layer at the SEI | bulk electrolyte interface. We perform numerical simulations of transport and reaction in the framework of this model and discuss distinct transport and reaction mechanisms depending on the rate constants for the molecule‐electron reactions. In particular, we show that an electron transport‐limited current regime exists, even if molecule transport inside the SEI is faster than electron transport. Based on the model results, we obtain good estimates for the transport coefficients of both electrons and molecules in SEIs formed at different potentials. Furthermore, we show that the transport and reaction model can be mapped onto a transmission‐line equivalent circuit.

## Experimental

All electrochemical experiments were carried out in a four‐electrode‐based generator‐collector setup as described in Ref. [36]. Sigradur‐G glassy carbon (HTW Hochtemperatur‐Werkstoffe GmbH) was used as the collector electrode (WE1 = first working electrode), which was contacted to a thermo‐block of a TSC Surface Cell (rhd instruments GmbH & Co. KG). Prior to use, the glassy carbon electrodes were polished in three steps employing diamond paste (Kemet International Ltd) with decreasing average particle diameters (3 μm, 1 μm, and 0.25 μm, respectively) and afterwards cleaned with HPLC grade acetone in an ultrasonic bath for three consecutive cycles. The area of the collector electrode in contact to the electrolyte was governed by an O‐ring (EPDM) with an inner diameter of 6 mm, yielding an active electrode area of 0.28 cm^2^. Compared to Ref. [36], the generator electrode (WE2 = second working electrode) consisted of glassy carbon instead of sputtered gold, resulting in a higher stability of the electrode surface. The generator electrode diameter is 4 mm, yielding an active electrode area of 0.13 cm^2^. The distance between generator and collector electrode, w , was determined from the bulk capacitance of the liquids *m*‐xylene and *n*‐dodecane in a parallel‐plate capacitor configuration of the two electrodes, as described in Section 1 of the Supporting Information. A value of μm was obtained. Lithium metal ribbons (Rockwood Lithium) were used as counter and reference electrode, respectively. The electrolyte (0.7 mL) was a 1 M solution of LiPF_6_ in a mixture of organocarbonates (volume ratio 3 : 3 : 4 of ethylene carbonate, ethyl methyl carbonate, and dimethyl carbonate; all solvents were purchased from Sigma Aldrich; H_2_O < 20 ppm) with additional 10 mM ferrocene (Acros Organics). The airtight cell was assembled in an argon‐filled glovebox and then removed from the glovebox. All measurements were performed outside the glovebox at room temperature (25 °C).

The SEI was formed in a potentiostatic mode at 0.1, 0.6 and 0.8 V vs. Li^+^/Li, respectively, applied over a time period of 50 hours. The electrolyte reduction current at the WE1 was measured continuously. Electrochemical impedance spectroscopy (EIS) and generator−collector experiments were performed every 4 hours. The impedance spectra were taken before starting the respective generator‐collector experiment. To avoid AC coupling, the generator (WE2) was set to open‐circuit conditions, and the impedance spectrum was recorded in a three‐electrode setup with the SEI‐covered glassy carbon electrode acting as working electrode. The spectra were analyzed in the elastance plane to extract the SEI thickness $d_{SEI}$ (see Section 2 of the Supporting Information). After recording the impedance spectrum, the generator electrode was reconnected to the circuit, and a potential of 3 V vs. Li^+^/Li (“off”‐state) was applied for 12 min., before switching the generator electrode to the “on”‐state (3.5 V vs. Li^+^/Li) for a time period of 300 s.

Based on new insights from the transport and reaction model, which will be explained in “Results and Discussion” section, the calculation of the “off”‐state and “on”‐state current density was modified compared to Krauss et al.^[36]^ For calculating the “off”‐state current density, the reduction current in the “off” state $I_{off}\left(t\right)$ was fitted by a linear function between $t-t_{start}=-300\;\;s$ and 0, and this fit was extrapolated to $t-t_{start}=300\;\;s$ . The extrapolated “off”‐state current $I_{off,\;extrapol}\left(t-t_{start}=300\;\;s\right)$ was then normalized by the area of the collector electrode to obtain the current density due to organocarbonate reduction:

(1) $$j_{carbonate}=\frac{I_{off,extrapol}\left(t-t_{start}=300\;\;s\right)}{A_{collector}}$$

Since the distance between generator and collector electrode is small compared to their diameters, we assume that the Fc^+^ ions produced at the generator electrode in the “on”‐state are reduced exclusively at a fraction of the surface area of the collector electrode, which is given by $A_{generator}=0.13\;{cm}^{2}$ . The results of COMSOL simulations shown in Section 4 of the Supporting Information justify this assumption. Furthermore, based on the results of a transport and reaction model, which will be discussed below, we assume that during the “on”‐state, electrolyte reduction does not contribute at all to the reduction current in this area. Consequently, we express the “on”‐state current density as a sum of a redox molecule reduction current inside the area $A_{generator}$ and an electrolyte reduction current in the remaining area $A_{collector}-A_{generator}$ . Electrolyte reduction in the remaining area should lead to the same current density as in the “off”‐state, namely $j_{carbonate}$ . Thus, the total current in the “on”‐state can be expressed as:

(2) $$I_{on}=j_{{Fc}^{+}}^{exp}\;A_{generator}+j_{carbonate}\;\left(A_{collector}-A_{generator}\right),$$

with $j_{{Fc}^{+}}^{exp}$ denoting the experimentally observed current density due to Fc^+^ ion reduction. Solving Eq. (2) for this current density yields:

(3) $$j_{{Fc}^{+}}^{exp}=\frac{I_{on}}{A_{generator}}-j_{carbonate}\;\frac{A_{collector}-A_{generator}}{A_{generator}}.$$

As shown in Ref. [36], $j_{{Fc}^{+}}^{exp}$ can be influenced by a Fc^+^ ion concentration drop in the bulk electrolyte from $c_{{Fc}^{+}}^{generator}$ to $c_{{Fc}^{+}}^{collector}$ in the case of poorly passivating SEIs. This is taken into account by calculating a corrected current density $j_{{Fc}^{+}}$ ,

(4) $$j_{{Fc}^{+}}=\left(1-\frac{w\;j_{{Fc}^{+}}^{exp}}{w\;j_{{Fc}^{+}}^{exp}+F\;c_{{Fc}^{+}}^{generator}\;D_{{Fc}^{+}}^{bulk}}\right)\;j_{{Fc}^{+}}^{exp},$$

which reflects exclusively the transport and reaction properties of the SEI.^[36]^ Since the SEI passivates well with regard to carbonate reduction already after short SEI formation times, this correction was not needed for $j_{carbonate}$ .

## Model Concept and Assumptions

### Transport and Reaction Model

**Figure 1:**
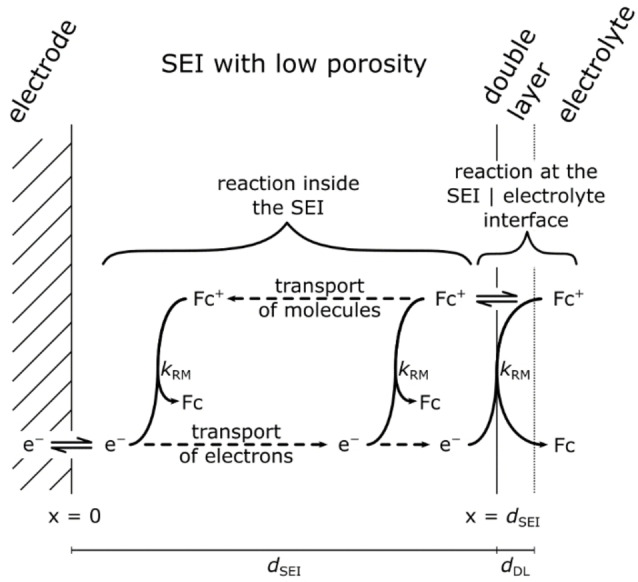
Schematic illustration of redox molecule transport, electron transport and reaction inside the inner SEI as well as at the SEI | bulk electrolyte interface. *k*_RM_ denotes the kinetic rate constant for the reaction of Fc^+^ ions and electrons. The transport and reaction of organocarbonate molecules, which is not shown here, is characterized by the same molecular diffusion coefficient, but by a lower kinetic rate constant *k*_M_. An outer SEI layer is neglected, since it is assumed that the outer layer is highly porous and does not contribute to passivation

We assume that the passivating properties of the SEI are caused by an inner layer with low porosity ϵ≪1 extending from x=0 to $x=d_{SEI}$ , see Figure 1. We neglect an outer SEI layer, since we assume that this outer layer is highly porous and does not contribute to passivation. Thus, at $x>d_{SEI}$ , the concentration of all molecular and ionic species is assumed to be identical to those in the bulk electrolyte. We assume that the pore space of the inner SEI is filled by the liquid electrolyte. The low porosity ϵ≪1 implies that the average concentration of a molecular species i close to $x=d_{SEI}$ , $c_{i}^{x=d_{SEI}}=\epsilon \;c_{i}^{bulk}$ , is much lower than its concentration in the bulk electrolyte $c_{i}^{bulk}$ . Furthermore, we assume that the diffusion coefficients D of all molecular species inside the pores of the inner SEI are identical.

The electron transfer at the glassy carbon | SEI interface at x=0 is assumed to be fast, so that the electron concentration $c_{eon}^{x=0}$ at this interface is given by the Nernst equation for a local equilibrium:

(5) $$c_{eon}^{x=0}\left(E\right)=c_{eon}^{x=0}\left(E=0\right)\;exp\left(-\frac{F\;E}{R\;T}\right).$$

The electrons are exclusively transported by diffusion characterized by a diffusion coefficient $D_{eon}$ . Electron migration is neglected due to the high Li^+^ concentration in the SEI.^[17]^

The reaction rate between molecular species and electrons is given by a rate equation, which is first order with respect to both molecules and electrons:

(6) $$\frac{dc_{i}\left(x\right)}{dt}=-k_{i}\;c_{i}\left(x\right)\;c_{eon}\left(x\right).$$

We assume that the rate constant of the redox molecules, $k_{RM}$ , is much higher than that of the organocarbonate molecules, $k_{M}$ , due to the much higher overpotential of the Fc^+^ ion reduction compared to the organocarbonate molecule reduction.^[36]^ This assumption is supported by quantum chemical calculations of the reorganization energy for the electron transfer reaction of ethylene carbonate molecules, yielding values in the range of 3.0–3.5 eV.^[43]^ Such high values should lead to very low rate constants for the organocarbonate reduction at low overpotentials, many orders of magnitude lower than the rate constant for the redox molecule reduction at high overpotentials.

The combination of transport and reaction leads to the following equation for the temporal evolution of the concentration profiles of molecules and electrons:

(7) $$\frac{dc_{i}\left(x\right)}{dt}=D\;\frac{d^{2}c_{i}\left(x\right)}{dx^{2}}-k_{i}\;c_{eon}\left(x\right)\;c_{i}\left(x\right)$$

and

(8) $$\frac{dc_{eon}\left(x\right)}{dt}=D_{eon}\;\frac{d^{2}c_{eon}\left(x\right)}{dx^{2}}-k_{i}\;c_{eon}\left(x\right)\;c_{i}\left(x\right).$$

In addition to the transport and reaction scenario inside the SEI, we assume that a heterogeneous reaction can take place at the SEI | bulk electrolyte interface, where electrons transported across the SEI react with molecules in the bulk electrolyte. This reaction is spatially limited to the thickness of the double layer at this interface, $d_{DL}$ , see Figure 1. The temporal evolution of the electron concentration at the SEI | bulk electrolyte interface can then be written as:

(9) $$\frac{dc_{eon}^{x=d_{SEI}}\;}{dt}=D_{eon}\frac{d^{2}c_{eon}^{x=d_{SEI}}}{dx^{2}}-k_{i}\;c_{eon}^{x=d_{SEI}}\;\left({c_{i}^{x=d_{SEI}}+c}_{i}^{bulk}\right).$$

In the stationary states, which will be discussed below, the time derivative of all concentrations is zero, i. e. $\frac{dc_{i}\left(x\right)}{dt}=0$ , $\frac{dc_{eon}\left(x\right)}{dt}=0$ , and $\frac{dc_{eon}^{x=d_{SEI}}\;}{dt}=0$ .

All model parameters are listed in Table 1.

**Table 1:** List of Symbols and Main Definitions.

symbol	
$c_{i}$	concentration of molecular species i ($M,\;mol\;{dm}^{-3}$ )
$c_{i}^{bulk}$	bulk concentration of molecular species i ($M,\;mol\;dm^{-3}$ )
$c_{i}^{x=d_{SEI}}$	concentration of molecular species i at the outermost SEI layer close to the SEI | bulk electrolyte interface ($M,\;mol\;{dm}^{-3}$ )
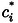	concentration of molecular species i in the SEI relative to its maximum concentration in the SEI, 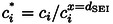
$c_{eon}$	concentration of electrons in the SEI ($M,\;mol\;dm^{-3}$ )
$c_{eon}^{x=0}$	concentration of electrons in the SEI at the electrode | SEI interface ($M,\;mol\;dm^{-3}$ )
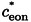	concentration of electrons in the SEI relative to their maximum concentration in the SEI, 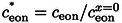
$c_{{Fc}^{+}}^{generator}$	concentration of ferrocenium ions at the generator electrode during the “on”‐state ($M,\;mol\;{dm}^{-3}$ )
$d_{SEI}$	thickness of the SEI (m )
$d_{DL}$	thickness of the electrochemical double layer (m )
$D_{i}^{bulk}$	bulk diffusion coefficient of molecular species i ($m^{2}\;s^{-1}$ )
D	diffusion coefficient of the molecular species in the SEI ($m^{2}\;s^{-1}$ )
$D_{eon}$	diffusion coefficient of electrons in the SEI ($m^{2}\;s^{-1}$ )
F	Faraday constant ($96485.332\;\;C\;{mol}^{-1}$ )
$J_{i}$	molecular flux of species i ($mol\;m^{-2}\;s^{-1}$ )
I	current (A )
$I_{off}$	total current of the collector electrode in the “off”‐state (A )
$I_{off,extrapol}$	extrapolated reduction current from the linear fit of the reduction current in the “off”‐state $I_{off}\left(t\right)$ fitted between $t-t_{start}=-300\;\;s$ and 0 (A )
$I_{on}$	total current of the collector electrode in the “on”‐state (A )
$j_{i}$	current density of molecular species i ($A\;{cm}^{-2}$ )
$j_{carbonate}$	current density of organocarbonate reduction ($A\;{cm}^{-2}$ )
$j_{{Fc}^{+}}$	current density of the ferrocenium ion reduction ($A\;{cm}^{-2}$ )
${\tilde{r}}_{eon}$	dimensionless transport resistance of the electrons in the SEI
${\tilde{r}}_{i}$	dimensionless transport resistance of molecular species i in the SEI
${\tilde{r}}_{CT}^{SEI}$	dimensionless charge‐transfer resistance inside the SEI
${\tilde{R}}_{CT}^{SEI}$	dimensionless charge‐transfer resistance of the reaction at the electrode | SEI interface
${\tilde{R}}_{CT}^{int}$	dimensionless charge‐transfer resistance of the reaction at the SEI | bulk electrolyte interface
E	electrode potential (V )
ϵ	porosity of the SEI, $\epsilon =c_{i}^{x=d_{SEI}}/c_{i}^{bulk}$ (dimensionless)
R	universal gas constant ($8.314\;\;J\;K^{-1}\;{mol}^{-1}$ )
T	temperature (K )
w	distance between generator and collector electrode (μm )
$k_{i}$	rate constant for the reaction of electrons with molecular species i ($m^{3}\;{mol}^{-1}\;s^{-1}$ )
$A_{generator}$	geometrical surface area of the generator electrode (${cm}^{2}$ )
$A_{collector}$	geometrical surface area of the collector electrode (${cm}^{2}$ )
$\chi_{i}$	passivation factor of the SEI with regard to molecular species i (dimensionless)
$\sigma_{eon}$	Onsager transport coefficient of the electrons in the SEI ($S\;{cm}^{-1})$
$\sigma_{carbonate}$	Onsager transport coefficient of the organocarbonate molecules in the SEI ($S\;{cm}^{-1})$
$\sigma_{{Fc}^{+}}$	Onsager transport coefficient of the Fc^+^ ions in the SEI ($S\;{cm}^{-1})$

### Dimensionless Parameters for Model Simulations

For the numerical model simulations, we define dimensionless parameters, which are listed in Table 2.

**Table 2:** Definition of dimensionless parameters for the numerical simulations.

parameter	dimensionless form
concentration of molecular species *i*	${\tilde{c}}_{i}=\frac{c_{i}}{c_{carbonate}^{bulk}}$
concentration of electrons inside the SEI	${\tilde{c}}_{eon}=\frac{c_{eon}}{c_{carbonate}^{bulk}}$
diffusion coefficient of all molecular species inside the SEI	$\tilde{D}=\frac{D}{D}=1$
diffusion coefficient of localized electrons inside the SEI	${\tilde{D}}_{eon}=\frac{D_{eon}}{D}$
spatial coordinate in x direction	$\tilde{x}=\frac{x}{d_{DL}}$
SEI thickness	${\tilde{d}}_{SEI}=\frac{d_{SEI}}{d_{DL}}$
thickness double layer	${\tilde{d}}_{DL}=\frac{d_{DL}}{d_{DL}}=1$
current density and flux	${\tilde{j}}_{i}=\frac{-j_{i}\;d_{DL}}{c_{carbonate}^{bulk}\;D}=\frac{J_{i}\;F\;d_{DL}}{c_{carbonate}^{bulk}\;D\;F\;}={\tilde{J}}_{i}$
rate constant	${\tilde{k}}_{i}=\frac{k_{i}\;{d_{DL}}^{2}\;c_{carbonate}^{bulk}}{D}$

The dimensionless current density in the stationary state was calculated by integrating Eq. (8) over the thickness of the SEI and Eq. (9) over the double layer thickness at the SEI | bulk electrolyte interface:

(10) $${\tilde{j}}_{i}={{\tilde{D}}_{eon}\left.\frac{d{\tilde{c}}_{eon}}{d\tilde{x}}\right|}_{\tilde{x}=0}={\tilde{k}}_{i}\left[\underset{\tilde{x}=0}{\int^{{\tilde{d}}_{SEI}}}{\tilde{c}}_{eon}\left(\tilde{x}\right)\;{\tilde{c}}_{i}\left(\tilde{x}\right)\;d\tilde{x}+{\tilde{c}}_{eon}^{\tilde{x}={\tilde{d}}_{SEI}}\;{\tilde{c}}_{i}^{bulk}\;{\tilde{d}}_{DL}\right]$$

### Transmission‐Line Model (TLM)

**Figure 2:**
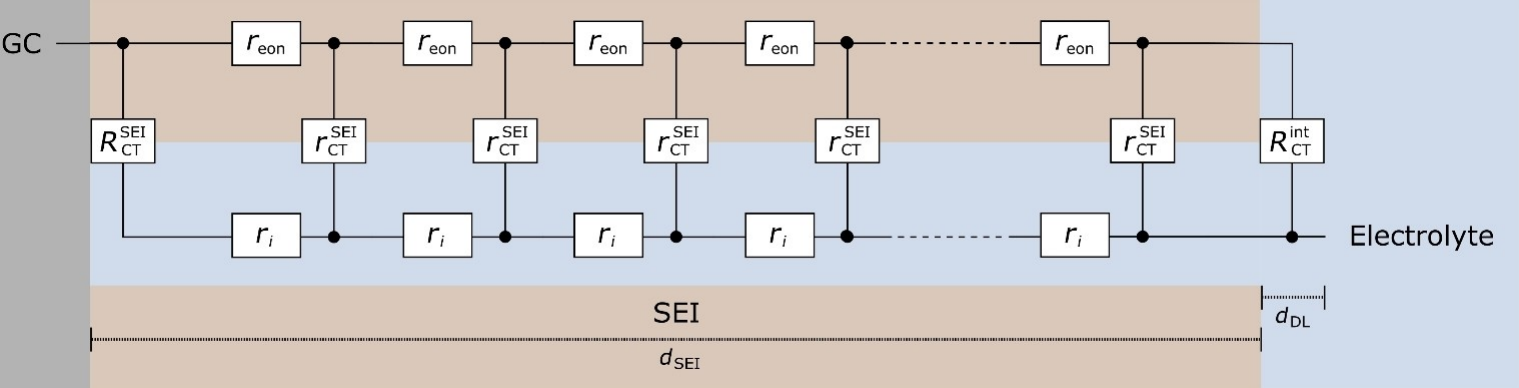
Z‐type transmission‐line model with P−Q conditions^[44]^ for the SEI. $d_{SEI}$ denotes the SEI thickness, $d_{DL}$ the double layer thickness, $r_{eon}$ an electron transport resistance, $r_{i}$ a transport resistance of the molecular species *i*, $r_{CT}^{SEI}$ a charge‐transfer resistance inside the SEI, $R_{CT}^{int}$ the charge‐transfer resistance at the SEI | bulk electrolyte interface, and $R_{CT}^{SEI}$ the charge‐transfer resistance at the electrode | SEI interface.

We compare the transport and reaction model simulations to a Z‐type transmission‐line model with P−Q conditions,^[44]^ which is illustrated in Figure 2. In this model, the diffusion of molecular species in the electrolyte‐filled pores of the SEI is described by the diffusion resistances $r_{i}.$ In dimensionless units, these diffusion resistances are given by:

(11) $${\tilde{r}}_{i}=\frac{1}{\epsilon \;{\tilde{c}}_{i}^{bulk}\;\tilde{D}}.$$

Electron diffusion is modeled by the electronic resistances $r_{eon}$ , which in dimensionless units are written as:

(12) $${\tilde{r}}_{eon}=\frac{1}{{\tilde{c}}_{eon}^{\tilde{x}=0}\;{\tilde{D}}_{eon}}.$$

The charge transfer reaction between molecules and electrons inside the SEI is described by the charge transfer resistances $r_{CT}^{SEI}$ , which in dimensionless units are given by:

(13) $${\tilde{r}}_{CT}^{SEI}=\frac{1}{{\tilde{k}}_{i}\;{\tilde{c}}_{eon}^{\tilde{x}=0}\;\epsilon \;{\tilde{c}}_{i}^{bulk}}.$$

The charge transfer resistance at the electrode | SEI interface,$\;R_{CT}^{SEI}$ , can be written in dimensionless units as:

(14) $${\tilde{R}}_{CT}^{SEI}=\frac{1}{{\tilde{k}}_{i}\;{\tilde{c}}_{eon}^{\tilde{x}=0}\;\epsilon \;{\tilde{c}}_{i}^{bulk}\;{\tilde{d}}_{DL}}.$$

The heterogeneous reaction at the SEI | bulk electrolyte interface is modeled by a charge‐transfer resistance $R_{CT}^{int}$ , which in dimensionless units is written as:

(15) $${\tilde{R}}_{CT}^{int}=\frac{1}{{\tilde{k}}_{i}\;{\tilde{c}}_{eon}^{\tilde{x}=0}\;{\tilde{c}}_{i}^{bulk}\;{\tilde{d}}_{DL}\;}.$$

In Eqs. (14) and (15), we use the same dimensionless double layer thickness at both interfaces. While this is an approximation, the decisive point is that ${\tilde{R}}_{CT}^{SEI}$ is much higher than ${\tilde{R}}_{CT}^{int}$ due to the low porosity of the SEI, ϵ .

The total resistance of this TLM circuit, given in Ref. [44], defines the inverse dimensionless current density in our SEI model:

(16) $$\frac{1}{{\tilde{j}}_{i}}=\frac{{\tilde{r}}_{i}\;{\tilde{r}}_{eon}}{{\tilde{r}}_{i}+{\tilde{r}}_{eon}}\;{\tilde{d}}_{SEI}+\frac{\sqrt{{\tilde{r}}_{CT}^{SEI}}}{{\left({\tilde{r}}_{i}+{\tilde{r}}_{eon}\right)}^{3/2}}\;\;\frac{\left[\begin{array}{c} \left({{\tilde{r}}_{i}}^{2}+{{\tilde{r}}_{eon}}^{2}\right)\;\cosh\beta +2\;{\tilde{r}}_{i}\;{\tilde{r}}_{eon}\\ +\left(\frac{{{\tilde{r}}_{i}}^{2}}{{\tilde{R}}_{CT}^{SEI}}+\frac{{{\tilde{r}}_{eon}}^{2}}{{\tilde{R}}_{CT}^{int}}\right)\;\sqrt{{\tilde{r}}_{CT}^{SEI}\;\left({\tilde{r}}_{i}+{\tilde{r}}_{eon}\right)}\;\sinh\beta \end{array}\right]}{\left[\begin{array}{c} \left(1+\frac{{\tilde{r}}_{CT}^{SEI}\left({\tilde{r}}_{i}+{\tilde{r}}_{eon}\right)}{{\tilde{R}}_{CT}^{SEI}\;{\tilde{R}}_{CT}^{int}}\right)\;\sinh\beta \\ +\left(\frac{1}{{\tilde{R}}_{CT}^{SEI}}+\frac{1}{{\tilde{R}}_{CT}^{int}}\right)\;\sqrt{{\tilde{r}}_{CT}^{SEI}\;\left({\tilde{r}}_{i}+{\tilde{r}}_{eon}\right)}\;\cosh\beta \end{array}\right]}$$

with

(17) $$\beta ={\tilde{d}}_{SEI}\sqrt{\frac{{\tilde{r}}_{i}+{\tilde{r}}_{eon}}{{\tilde{r}}_{CT}^{SEI}}}.$$

## Results and Discussion

### Experimental Results

**Figure 3:**
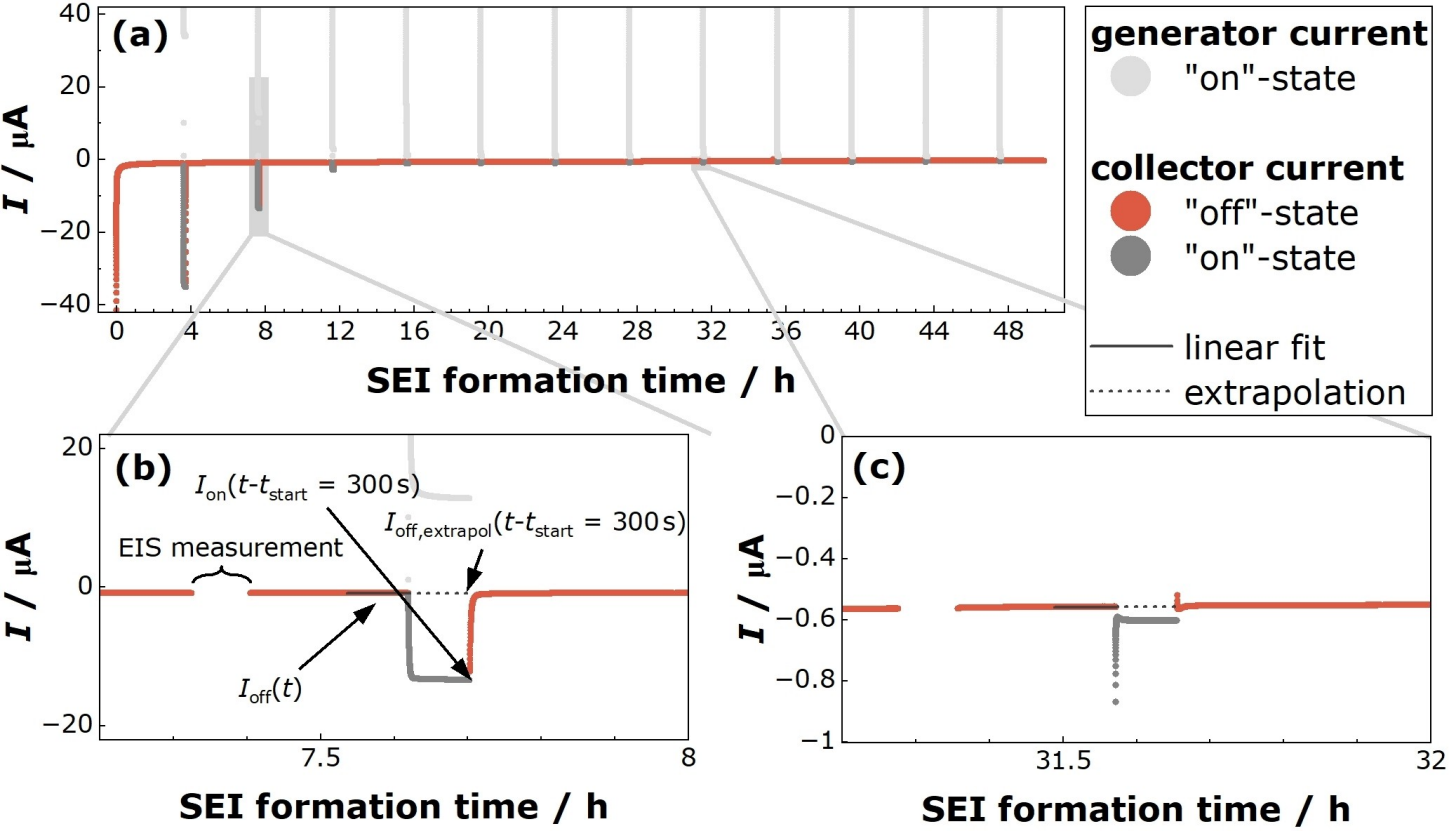
Measured currents during SEI formation at 0.6 V vs. Li^+^/Li as well as during the generator‐collector experiments. (a) Complete time window of the experiment. (b) and (c) Zoom into currents measured close to 8 hours and close to 32 hours, respectively. The reduction currents in the “on”‐ and “off”‐state are marked with arrows. $I_{off,extrapol}\left(t-t_{start}=300\;\;s\right)$ and $I_{on}\left(t-t_{start}=300\;\;s\right)$ are marked exemplarily in (b).

In Figure 3(a), we show the measured currents during potentiostatic SEI formation at 0.6 V vs. Li^+^/Li as well as during the generator‐collector experiments. Results for 0.1 V and 0.8 V are shown in Figure S4 and S6 of the Supporting Information. While results for 0.8 V were already presented in Ref. [36] we repeated the measurements, since we used a slightly modified experimental setup. As seen from Figure 3(a), the electrolyte reduction current in the “off”‐state, $I_{off}$ , as well as the current measured in the “on”‐state, $I_{on}$ , decrease strongly with increasing SEI formation time. In Figure 3(b) and (c), the time‐dependent currents during a generator‐collector experiment on SEI formation time scales of about 8 hours and about 32 hours, respectively, are shown in more detail. The dotted lines show the fitted “off”‐state current and its extrapolation into the “on”‐state time regime. The impedance spectra recorded before the respective generator‐collector experiment and the analysis in the elastance plane to extract the average SEI thickness $d_{SEI}$ are shown in Section 2 of the Supporting Information.

It is important to note that after switching the generator electrode from the “on”‐state to the “off”‐state, the Fc^+^ ions in the bulk electrolyte are reduced at the generator electrode. As shown in Figure S7 of the Supporting Information, about 1 hour after the “on”→“off” switching, the reductive charge at the generator electrode is virtually identical to the oxidative charge in the “on”‐state. Thus, virtually all Fc^+^ ions have been removed from the electrolyte, and the subsequent generator‐collector experiment is not influenced by residual Fc^+^ ions.

**Figure 4:**
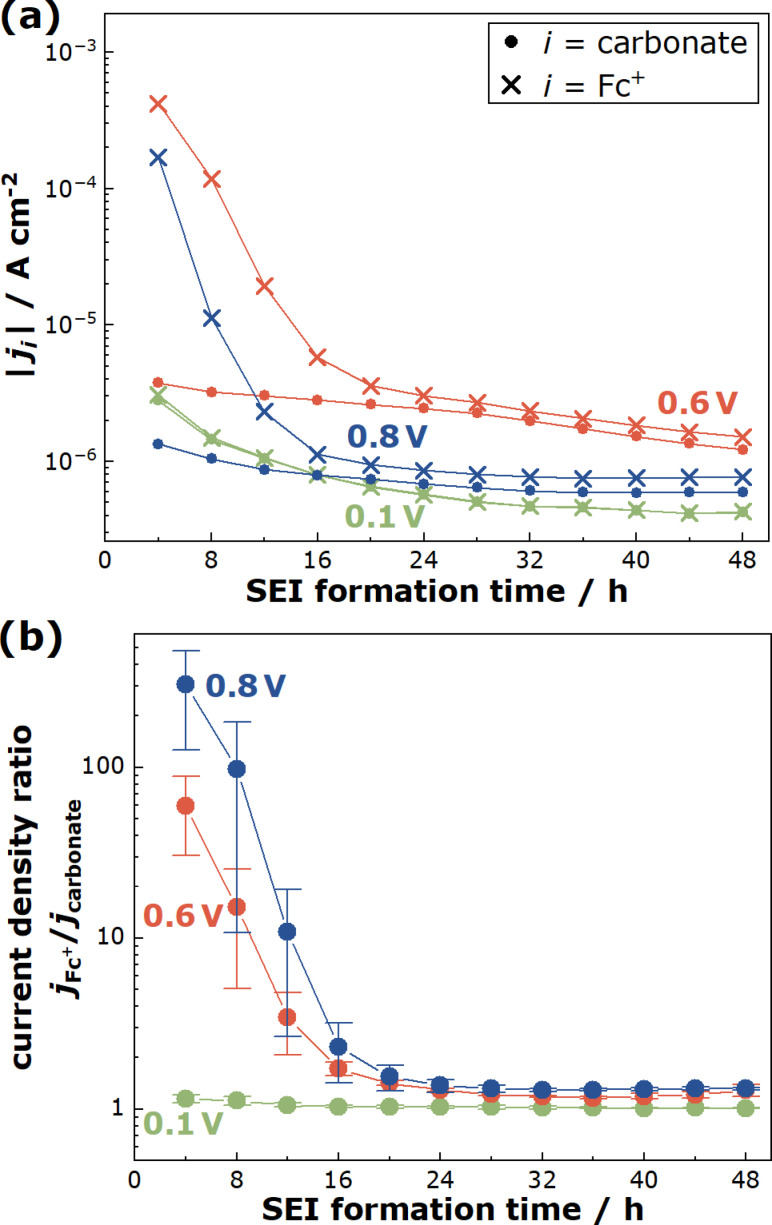
(a) Current densities $j_{carbonate}$ , calculated from Eq. (1), and $j_{{Fc}^{+}}$ , calculated from Eq. (4), plotted versus the SEI formation time. (b) Current density ratio *j*_FC_^+^/*j*_carbonate_ plotted versus the SEI formation time.

In Figure 4(a), we plot the current densities $j_{carbonate}$ and $j_{{Fc}^{+}}$ obtained from Eqs. (1) and (4) versus the SEI formation time at different SEI formation potentials. The ratio of both current densities $j_{{Fc}^{+}}/j_{carbonate}$ is plotted versus the SEI formation time in Figure 4(b). In the case of SEI formation at 0.8 V vs. Li^+^/Li, the current density ratio is in the range of 300 at short SEI formation times and drops to 1.3 at long SEI formation times. In the case of 0.6 V, the current density ratio drops from about 60 to 1.2, while in the case of 0.1 V, the ratio drops from 1.15 to 1.01. This shows that the SEI transport and passivation properties depend strongly on the SEI formation potential.

As explained in detail in Ref. [36] we define passivation factors for the electrolyte and for the Fc^+^ ion reduction, respectively, by comparing the measured current densities to the current densities for a hypothetical SEI with the same thickness as the real SEI, but with the transport properties of the bulk electrolyte:

(18) $$\chi_{carbonate}=-\frac{z\;F\;c_{carbonate}^{bulk}\;D_{carbonate}^{bulk}}{j_{carbonate}\;d_{SEI}},$$

and

(19) $$\chi_{{Fc}^{+}}=-\frac{F\;c_{{Fc}^{+}}^{generator}\;D_{{Fc}^{+}}^{bulk}}{j_{{Fc}^{+}}\;d_{SEI}}.$$

Here, $c_{carbonate}^{bulk}$ and $D_{carbonate}^{bulk}$ denote the bulk concentration and the bulk diffusion coefficient of organocarbonate molecules with $c_{carbonate}^{bulk}=12\;\;mol\;{dm}^{-3}$ ^[36]^ and $D_{carbonate\;}^{bulk}=3.5\;\cdot \;10^{-6}{cm}^{2}s^{-1}$ ,^[45]^ respectively. Since one‐electron and two‐electron reduction reactions of organocarbonate molecules have been proposed,[^13^, ^46^, ^47^, ^48^, ^49^, ^50^] we assume an average number of transferred electrons of z=1.5 . $c_{{Fc}^{+}}^{generator}$ denotes the concentration of Fc^+^ ions at the generator electrode, which is identical to the concentration of ferrocene in the bulk electrolyte: $c_{{Fc}^{+}}^{generator}=c_{Fc}^{bulk}=10\;\;mmol\;{dm}^{-3}$ . The bulk diffusion coefficient of the Fc^+^ ions, $D_{{Fc}^{+}}^{bulk}$ , was assumed to be identical to that of ferrocene $D_{Fc}^{bulk}=3.76\cdot 10^{-6}\;{cm}^{2}s^{-1}$ .^[36]^

**Figure 5:**
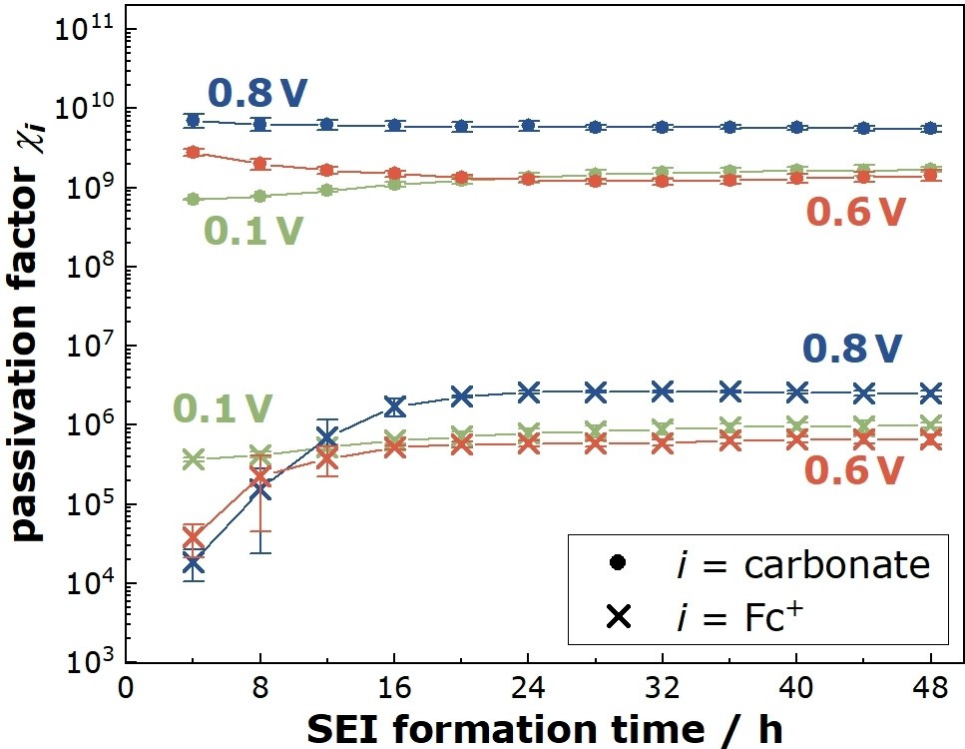
Temporal evolution of the passivation factors for organocarbonate molecules, *χ*_carbonate_, and for Fc^+^ ions, *χ*_FC_^+^, during the SEI formation at the potentials 0.1 V, 0.6 V, and 0.8 V vs. Li^+^/Li, respectively.

In Figure 5, the passivation factors $\chi_{carbonate}$ and $\chi_{{Fc}^{+}}$ are plotted versus the SEI formation time for all three formation potentials. The passivation factor $\chi_{carbonate}$ is virtually constant at formation times of 4 hours and longer. $\chi_{carbonate}$ is about 8 ⋅ 10^9^ at 0.8 V and close to $10^{9}$ at 0.6 V and 0.1 V. At 0.8 V and 0.6 V, the passivation factor of the redox molecules $\chi_{{Fc}^{+}}$ increases from values in the range $10^{4}-10^{5}$ at 4 hours to values in the range of $10^{6}$ at 48 hours. In contrast, at 0.1 V and for SEI formation times of 4 hours and longer, $\chi_{{Fc}^{+}}\;$ is virtually independent of the SEI formation time.

### Model Results

**Figure 6:**
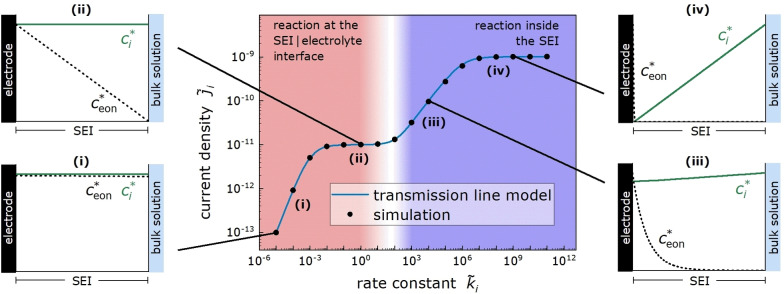
Dimensionless reduction current density ${\tilde{j}}_{i}$ of a molecular species *i* with ${\tilde{c}}_{i}^{bulk}=1$ plotted versus the dimensionless rate constant ${\tilde{k}}_{i}$ . The transport coefficient of the molecular species *i*, $\tilde{D}\;\epsilon \;{\tilde{c}}_{i}^{bulk}$ =10^−7^, is 100 times higher than the transport coefficient of the electrons, ${\tilde{D}}_{eon}\;{\tilde{c}}_{eon}^{\tilde{x}=0}$ =10^−9^. The red background color marks ${\tilde{k}}_{i}$ ‐regions with more than 99 % of the reaction taking place at the SEI | bulk electrolyte interface, while the violet background color marks ${\tilde{k}}_{i}$ ‐regions with more than 99 % of the reaction taking place inside the SEI. In (i) – (iv), typical molecule and electron concentration profiles in different transport and reaction regimes are shown. The plotted concentrations 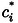 and 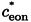 denote the simulated dimensionless concentrations ${\tilde{c}}_{i}$ and ${\tilde{c}}_{eon}$ relative to their maximum concentration inside the SEI ${\tilde{c}}_{i}^{\tilde{x}={\tilde{d}}_{SEI}}$ and ${\tilde{c}}_{eon}^{\tilde{x}=0}$ , respectively.

First, we show results of a simulation, in which the transport of the molecular species i in the SEI is faster than the electron transport. For this simulation, we chose the following parameters: ${\epsilon =10}^{-7},$ ${\tilde{c}}_{i}^{bulk}=1.0,$ ${\tilde{c}}_{eon}^{\tilde{x}=0}=10^{-8},$ $\tilde{D}=1.0,$ ${\tilde{D}}_{eon}=0.1$ and ${\tilde{d}}_{SEI}=100.$ These parameters lead to a transport coefficient of the molecular species $\tilde{D}\;\epsilon \;{\tilde{c}}_{i}^{bulk}=10^{-7}$ compared to an electron transport coefficient of ${\tilde{D}}_{eon}\;{\tilde{c}}_{eon}^{\tilde{x}=0}=10^{-9}.$ In Figure 6, we plot the obtained dimensionless current density ${\tilde{j}}_{i}$ versus the dimensionless rate constant ${\tilde{k}}_{i}$ . The data points are results from transport and reaction simulations using Eqs. (7) – (10), while the blue line are results from the TLM Eq. (16). There is excellent agreement between simulation results and TLM results, showing that the transport and reaction model is equivalent to the TLM.

Depending on the rate constant ${\tilde{k}}_{i}$ , four distinct transport and reaction regimes exist:

*Regime (i)* At very low values of ${\tilde{k}}_{i}$ , the reduction current is limited by the charge transfer rate of the molecule‐electron reaction at the SEI | bulk electrolyte interface. Accordingly, there is no electron concentration drop inside the SEI, as illustrated in Figure 6(i). As shown in Section 5 of the Supporting Information, the dimensionless current density is given by:

(20) $${\tilde{j}}_{i}={\tilde{k}}_{i}\;{\tilde{c}}_{i}^{bulk}\;{\tilde{c}}_{eon}^{\tilde{x}=0}{\;\tilde{d}}_{DL}.$$

Due to the much higher concentration of molecules in the bulk electrolyte, the reaction at the SEI | bulk electrolyte interface is faster than inside the SEI.

*Regime (ii)* With increasing rate constant ${\tilde{k}}_{i}$ , electron transport across the SEI becomes limiting, and the electron concentration drops to almost zero at $\tilde{x}={\tilde{d}}_{SEI}$ . As shown in Section 5 of the Supporting Information, the dimensionless current density is then given by:

(21) $${\tilde{j}}_{i}=\frac{{\tilde{D}}_{eon}\;{\tilde{c}}_{eon}^{\tilde{x}=0}}{{\tilde{d}}_{SEI}}.$$

Also in this regime, the reaction at the reaction at the SEI | bulk electrolyte interface is faster than inside the SEI.

*Regime (iii)* With increasing rate constant ${\tilde{k}}_{i}$ , the reaction shifts from the SEI | bulk electrolyte interface into the SEI. In Figure 6, this shift is illustrated by a color coding from red to blue. In regime (iii), the reduction current is limited by electron transport and by the charge transfer reaction inside the SEI, see Figure 6(iii). The charge transfer reaction leads to curved concentration profiles of both molecules and electrons inside the SEI according to Eqs. (7) and (8). As shown in Section 5 of the Supporting Information, the dimensionless current density is then given by:

(22) $${\tilde{j}}_{i}={\tilde{c}}_{eon}^{\tilde{x}=0}\;\sqrt{{{\tilde{k}}_{i}\;\tilde{c}}_{i}^{bulk}\;\epsilon \;{\tilde{D}}_{eon}}.$$

*Regime (iv)* At very high values of ${\tilde{k}}_{i}$ , electron and molecule transport in the SEI govern the reduction current. Since the electron transport is slower than the molecule transport, the electron and molecule profiles intersect close to the electrode | SEI interface, where the reaction takes place, see Figure 6(iv). As shown in Section 5 of the Supporting Information, the dimensionless current density is given by:

(23) $${\tilde{j}}_{i}=\frac{\tilde{D}\;\epsilon \;{\tilde{c}}_{i}^{bulk}+{\tilde{D}}_{eon}\;{\tilde{c}}_{eon}^{\tilde{x}=0}}{{\tilde{d}}_{SEI}}.$$

**Figure 7:**
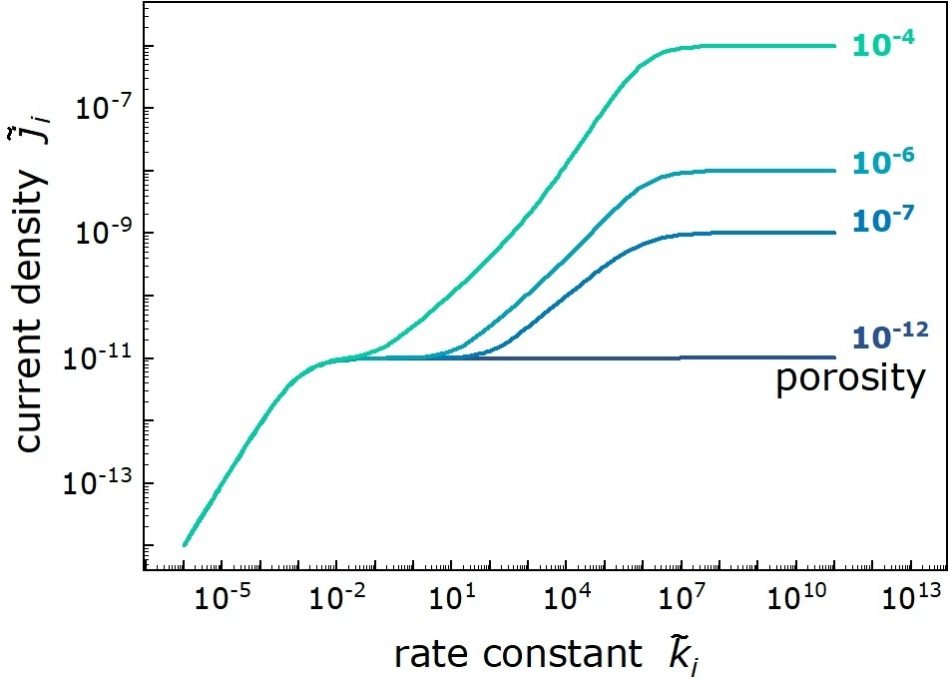
Dimensionless current density ${\tilde{j}}_{i}$ of a molecular species i with ${\tilde{c}}_{i}^{bulk}=1$ plotted versus the dimensionless rate constant ${\tilde{k}}_{i}$ at different SEI porosities of ε=10^−4^, 10^−6^, 10^−7^, and 10^−12^, respectively.

In Figure 7, we show ${\tilde{j}}_{i}\left({\tilde{k}}_{i}\right)$ results for the same simulation parameters, except that the porosity of the SEI was varied between ${\epsilon =10}^{-4}$ and ${\epsilon =10}^{-12}$ . As seen from Figure 7, the value of the porosity has no influence on the current density in regimes (i) and (ii). This is plausible, since in these regimes, the reaction rate at the SEI | bulk electrolyte interface and the electron transport across the SEI, respectively, are the limiting factors, which are not influenced by the porosity, see Eqs. (20) and (21). In contrast, the current density in regimes (iii) and (iv) is dependent on the porosity, since the reaction takes place inside the SEI and its rate is influenced by the molecule concentration in the SEI and thus by the porosity of the SEI, see Eqs. (22) and (23). As seen from Figure 7, regime (iii) is not detectable anymore at a porosity of ${\epsilon =10}^{-12}.$ In the case of this very low porosity, the molecule transport coefficient $\tilde{D}\;\epsilon \;{\tilde{c}}_{i}^{bulk}=10^{-12}$ becomes much smaller than the electron transport coefficient ${\tilde{D}}_{eon}\;{\tilde{c}}_{eon}^{\tilde{x}=0}=10^{-9}$ . Consequently, the dimensionless current density in regime (iv) ${\tilde{j}}_{i}=\frac{\tilde{D}\;\epsilon \;{\tilde{c}}_{i}^{bulk}+{\tilde{D}}_{eon}\;{\tilde{c}}_{eon}^{\tilde{x}=0}}{{\tilde{d}}_{SEI}}\;$ is virtually identical to the dimensionless current density in regime (ii) ${\tilde{j}}_{i}=\frac{{\tilde{D}}_{eon}\;{\tilde{c}}_{eon}^{x=0}}{{\tilde{d}}_{SEI}}$ .

Next we compare the model results to the experimental results. Since the passivation factor of the SEI with regard to organocarbonate reduction is virtually independent of the SEI formation time, the transport and reaction mechanism must be independent of the SEI porosity, i. e. the mechanism must be in regime (i) or (ii). The experimentally observed approximate $1/\sqrt{t}$ dependence of the current density^[17]^ gives strong indication that the organocarbonate reduction is transport‐limited, i. e. the mechanism is in regime (ii). Furthermore, it is evident that a current density ratio $j_{{Fc}^{+}}/j_{carbonate}>1$ , which drops with increasing SEI formation time, can only then be measured, if the transport and reaction mechanism for the redox molecules is in the porosity‐dependent regimes (iii) or (iv). This is plausible, since the reaction rate of the redox molecules $k_{{Fc}^{+}}$ is expected to be much higher than the reaction rate of the organocarbonate molecules$\;k_{carbonate}$ . Thus, we emphasize that in the framework of our model, the assumption of a time‐dependent SEI porosity is the only possibility to explain both the observed current density ratio (Fig. 4(b)) and an SEI thickness increasing with time (Fig. S3).

In the following, we consider three model cases depending on the SEI porosity: (I) redox molecule transport is much faster than electron transport, (II) redox molecule transport and electron transport are equally fast and (III) redox molecule transport is slower than electron transport.

**Figure 8:**
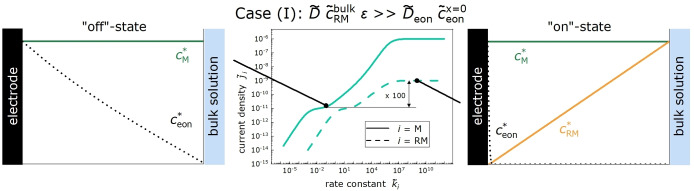
Dimensionless current densities ${\tilde{j}}_{i}$ of the molecular species i= M and RM for case (I) with ${\tilde{c}}_{M}^{bulk}=1$ and ${\tilde{c}}_{RM}^{bulk}=10^{-3}$ plotted versus the dimensionless rate constant ${\tilde{k}}_{i}$ . The SEI exhibits a porosity of $\epsilon =10^{-4}$ . The insets show the concentration profiles of electrons and molecules in the “off”‐state (left), for ${\tilde{k}}_{M}=0.1$ , and “on”‐state, for ${\tilde{k}}_{M}=0.1$ and ${\tilde{k}}_{RM}=10^{9}$ . The plotted concentrations 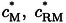 and 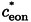 denote the stationary dimensionless concentration ${\tilde{c}}_{M}$ , ${\tilde{c}}_{RM}$ and ${\tilde{c}}_{eon}$ relative to their maximum dimensionless concentration inside the SEI ${\tilde{c}}_{M}^{\tilde{x}={\tilde{d}}_{SEI}}$ , ${\tilde{c}}_{RM}^{\tilde{x}={\tilde{d}}_{SEI}}$ and ${\tilde{c}}_{eon}^{\tilde{x}=0}$ , respectively.

Case (I) is relevant at relatively high SEI porosities. When we keep the parameters considered above and choose a dimensionless bulk redox molecule concentration ${\tilde{c}}_{RM}^{bulk}=10^{-3},$ which is three orders of magnitude lower than the dimensionless bulk organocarbonate concentration, and vary the porosity, case (I) is obtained for $\epsilon =10^{-4}$ . In this case, the electron transport coefficient ${\tilde{D}}_{eon}{\;\tilde{c}}_{eon}^{\tilde{x}=0}=10^{-9}$ is two orders of magnitude lower than the redox molecule transport coefficient $\tilde{D}\;\epsilon \;{\tilde{c}}_{RM}^{bulk}=10^{-7}$ . In Figure 8, we show plots of ${\tilde{J}}_{i}$ versus the rate constant, ${\tilde{k}}_{i},$ for both the organocarbonate molecules and the redox molecules. We note that due to the 10^3^ times lower concentration of the redox molecules in the bulk electrolyte, the redox molecule transport‐limited current in regime (iv) is 10^3^ times lower (dashed line) than the organocarbonate transport‐limited plateau (solid line) in the same regime.

When ${\tilde{k}}_{RM}$ for the redox molecules is at least eight orders of magnitude higher than ${\tilde{k}}_{M}$ for the organocarbonate molecules, the redox molecule reduction is determined by the transport coefficient $\tilde{D}\;\epsilon \;{\tilde{c}}_{RM}^{bulk}=10^{-7},$ while the organocarbonate molecule reduction is determined by the electron transport coefficient ${\tilde{D}}_{eon}{\;\tilde{c}}_{eon}^{\tilde{x}=0}=10^{-9}$ , resulting in a current density ratio $j_{RM}/j_{M}=100.$ A ratio of ${\tilde{k}}_{RM}/{\tilde{k}}_{M}\geq 10^{8}$ seems likely in view of the high reorganization energy for the electron transfer reaction of ethylene carbonate molecules.^[43]^ The corresponding concentration profiles in the “off”‐state and in the “on”‐state are shown in the insets of Figure 8. We note that the plotted concentrations 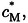 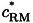 and 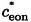 denote the stationary dimensionless concentration ${\tilde{c}}_{M}$ , ${\tilde{c}}_{RM}$ and ${\tilde{c}}_{eon}$ relative to their maximum concentration inside the SEI ${\tilde{c}}_{M}^{\tilde{x}={\tilde{d}}_{SEI}}$ , ${\tilde{c}}_{RM}^{\tilde{x}={\tilde{d}}_{SEI}}$ and ${\tilde{c}}_{eon}^{\tilde{x}=0}$ , respectively. The strong drop of the electron concentration profile at the electrode | SEI interface in the “on”‐state implies that the organocarbonate molecule reduction current in this state is negligible as compared to the redox molecule reduction current, since no electrons reach the SEI | bulk electrolyte interface.

**Figure 9:**
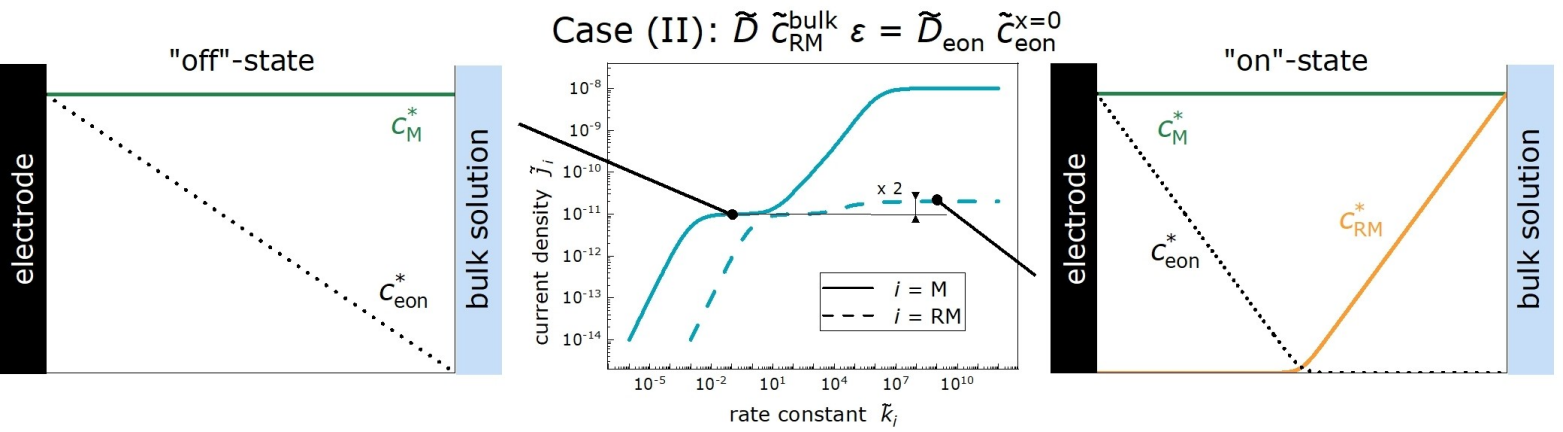
Dimensionless current densities ${\tilde{j}}_{i}$ of the molecular species i= M and RM for Case (II) with ${\tilde{c}}_{M}^{bulk}=1$ and ${\tilde{c}}_{RM}^{bulk}=10^{-3}$ plotted versus the dimensionless rate constant ${\tilde{k}}_{i}$ . The SEI exhibits a porosity of $\epsilon =10^{-6}$ . The insets show the concentration profiles of electrons and molecules in the “off”‐state (left), for ${\tilde{k}}_{M}=0.1$ , and “on”‐state (right), for ${\tilde{k}}_{M}=0.1$ and ${\tilde{k}}_{RM}=10^{9}$ . The plotted concentrations 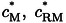 and 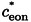 denote their simulated dimensionless concentration ${\tilde{c}}_{M}$ , ${\tilde{c}}_{RM}$ and ${\tilde{c}}_{eon}$ relative to their maximum dimensionless concentration inside the SEI ${\tilde{c}}_{M}^{\tilde{x}={\tilde{d}}_{SEI}}$ , ${\tilde{c}}_{RM}^{\tilde{x}={\tilde{d}}_{SEI}}$ and ${\tilde{c}}_{eon}^{\tilde{x}=0}$ , respectively.

Case (II) is relevant for intermediate SEI porosities, see Figure 9. For the parameters chosen here, $\epsilon =10^{-6}\;$ results in ${\tilde{D}}_{eon}{\;\tilde{c}}_{eon}^{\tilde{x}=0}=10^{-9}=\tilde{D}\;\epsilon \;{\tilde{c}}_{RM}^{bulk}$ . Due to the identical transport coefficients of redox molecules and electrons, the reaction takes place mainly in the center of the SEI, and the dimensionless current density is, to a good approximation given, by ${\tilde{j}}_{RM}=\frac{\tilde{D}\;\epsilon \;{\tilde{c}}_{RM}^{bulk}+{\tilde{D}}_{eon}\;{\tilde{c}}_{eon}^{\tilde{x}=0}}{{\tilde{d}}_{SEI}}$ , which is approximately twice as high as the “off”‐state current density ${\tilde{j}}_{M}=\frac{{\tilde{D}}_{eon}\;{\tilde{c}}_{eon}^{\tilde{x}=0}}{{\tilde{d}}_{SEI}}$ . In this case, the electron concentration drops to virtually zero around ${\tilde{x}=\tilde{d}}_{SEI}/2$ , so that virtually no electrons reach the SEI | bulk electrolyte interface. Consequently, the organocarbonate molecule reduction current in the “on”‐state is negligible.

**Figure 10:**
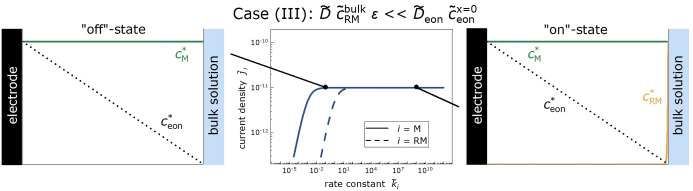
Dimensionless current densities ${\tilde{j}}_{i}$ of the molecular species i= M and RM for Case (III) with ${\tilde{c}}_{M}^{bulk}=1$ and ${\tilde{c}}_{RM}^{bulk}=10^{-3}$ plotted versus the dimensionless rate constant ${\tilde{k}}_{i}$ . The SEI exhibits a porosity of $\epsilon =10^{-12}$ . The insets show the concentration profiles of electrons and molecules in the “off”‐state (left), for ${\tilde{k}}_{M}=0.1$ , and “on”‐state (right), for ${\tilde{k}}_{M}=0.1$ and ${\tilde{k}}_{RM}=10^{9}$ . The plotted concentrations 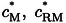 and 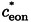 denote their simulated dimensionless concentration ${\tilde{c}}_{M}$ , ${\tilde{c}}_{RM}$ and ${\tilde{c}}_{eon}$ relative to their maximum dimensionless concentration inside the SEI ${\tilde{c}}_{M}^{\tilde{x}={\tilde{d}}_{SEI}}$ , ${\tilde{c}}_{RM}^{\tilde{x}={\tilde{d}}_{SEI}}$ and ${\tilde{c}}_{eon}^{\tilde{x}=0}$ , respectively.

Case (III) is relevant for very low SEI porosities. As an example, we show in Fig. 10 results for $\epsilon =10^{-12}$ implying that ${\tilde{D}}_{eon}{\;\tilde{c}}_{eon}^{\tilde{x}=0}\gg \tilde{D}\;\epsilon \;{\tilde{c}}_{RM}^{bulk}$ . In this case, ${\tilde{j}}_{RM}=\frac{\tilde{D}\;\epsilon \;{\tilde{c}}_{RM}^{bulk}+{\tilde{D}}_{eon}\;{\tilde{c}}_{eon}^{\tilde{x}=0}}{{\tilde{d}}_{SEI}}$ is virtually identical to the “off”‐state current density ${\tilde{j}}_{M}=\frac{{\tilde{D}}_{eon}\;{\tilde{c}}_{eon}^{\tilde{x}=0}}{{\tilde{d}}_{SEI}}$ , see Figure 10, and the Fc^+^ ion reduction takes place close to the SEI | bulk electrolyte interface. Also in this case, the organocarbonate current in the “on”‐state is negligible, since the electrons reaching the SEI | bulk electrolyte interface reduce the Fc^+^ ions with a probability proportional to ${{\tilde{k}}_{RM}\;\tilde{c}}_{RM}^{bulk}$ and the organocarbonate molecules with a much lower probability proportional to ${{\tilde{k}}_{M}\;\tilde{c}}_{M}^{bulk}.$

Considering all three cases (I), (II), and (III), we conclude that independent of the SEI porosity, measurements of the current densities $j_{RM}$ and $j_{M}$ yield information on the transport coefficients of redox molecules and electrons, respectively, in the SEI.

### Influence of SEI Formation Potential on Electron and Redox Molecule Transport

As discussed above, the organocarbonate reduction is governed by electron transport across the SEI. In this case, the transport coefficient of the electrons $c_{eon}^{x=0}\;D_{eon}$ , can be calculated from the long‐time limit of the passivation factor $\chi_{carbonate}\left(t\right)$ :^[36]^

(24) $$\underset{t\to \infty }{lim}\;\chi_{carbonate}\left(t\right)=\frac{c_{carbonate}^{bulk}\;D_{carbonate}^{bulk}}{c_{eon}^{x=0}\;D_{eon}}$$

This transport coefficient $c_{eon}^{x=0}\;D_{eon}$ can be converted into an Onsager transport coefficient via the Nernst‐Einstein relation, see Section 6 of the Supporting Information:

(25) $$\sigma_{eon}=\frac{F^{2}}{R\;T}\;c_{eon}^{x=0}\;D_{eon}$$

**Figure 11:**
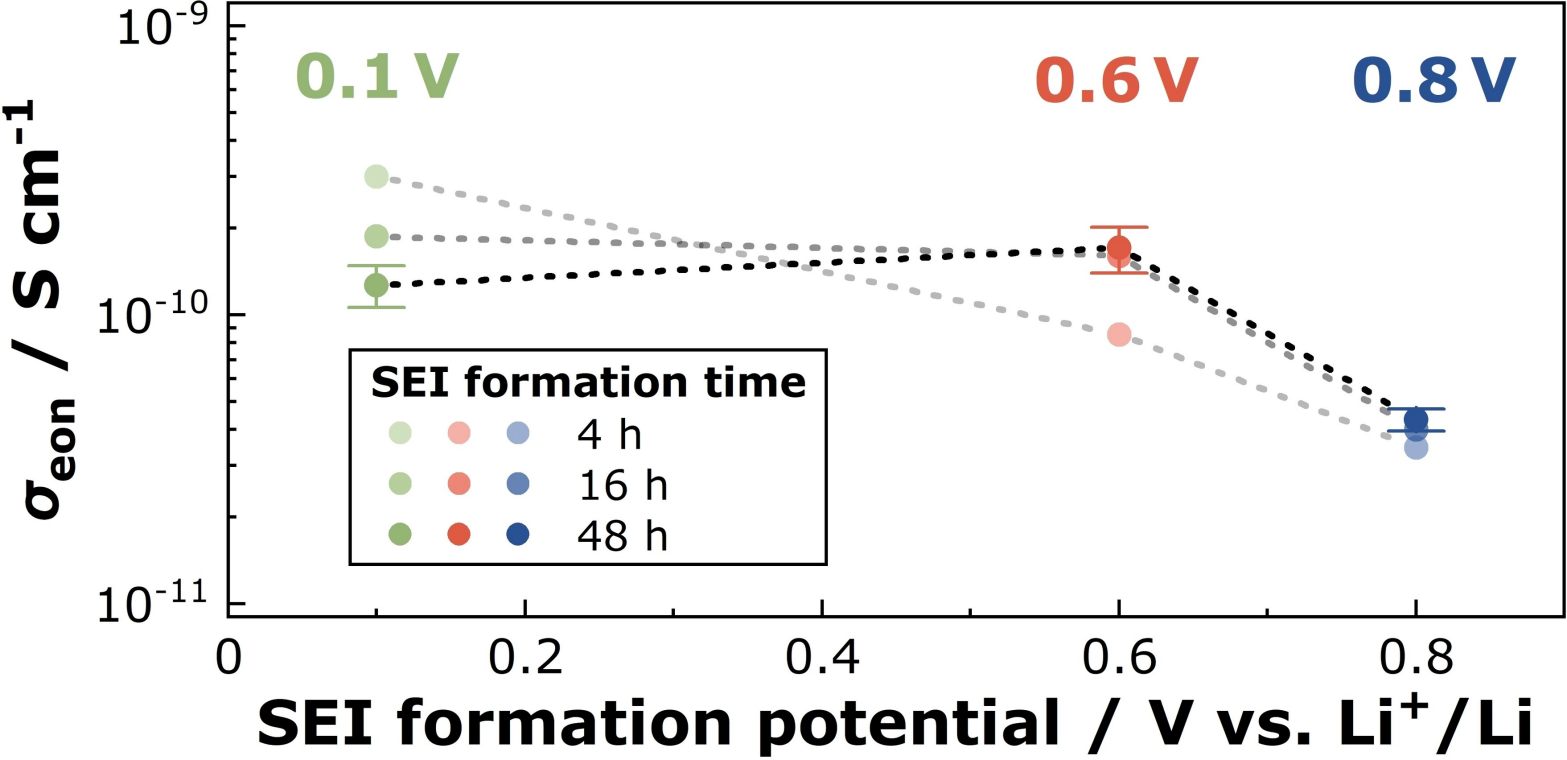
Experimentally determined Onsager transport coefficient of the electrons, $\sigma_{eon}$ , plotted versus the SEI formation potential for different SEI formation times of 4 h, 16 h and 48 h, respectively.

In Figure 11, the Onsager transport coefficient of the electrons is plotted versus the SEI formation potential for different SEI formation times. At a short SEI formation time of 4 h, the Onsager transport coefficient increases in order $\sigma_{eon}$ (0.8 V)=$3.5\cdot 10^{-11}\;S\;{cm}^{-1}$ $<\;\sigma_{eon}$ (0.6 V)=$8.5\cdot 10^{-11}\;S\;{cm}^{-1}\;<$ $\sigma_{eon}$ (0.1 V)=$3.0\cdot 10^{-10}\;S\;{cm}^{-1}$ . The high Onsager transport coefficient at 0.1 V should be the origin of the fast short‐time SEI growth at 0.1 V as shown in Figure S3 of the Supporting Information. On the other hand, the long‐time SEI growth is fastest at 0.6 V, see Figure S3, and this observation is in good agreement with the maximum of $\sigma_{eon}$ (0.6 V)=$1.7\cdot 10^{-10}\;S\;{cm}^{-1}$ at 48 h SEI formation time.

The maximum of the Onsager transport coefficient at 0.6 V and 48 hours SEI formation time suggests an influence of two opposing factors on *σ*_eon_. The validity of the Nernst equation for the local equilibrium at the electrode | SEI interface, see Eq. (5), implies an increasing electron concentration $c_{eon}^{x=0}$ with decreasing SEI formation potential. Consequently, the diffusion coefficient of the electrons *D*_eon_ must decrease with decreasing electrode potential. Our results illustrated in Figure S3 and Figure 11 suggest that the passivation properties of our model‐type SEIs differ from SEIs on the graphite anode of commercial full lithium‐ion battery cells, as probed by calendar aging experiments.[^51^, ^52^] This gives strong indication that a transport model created for a specific SEI cannot be generalized, but it is important to carry out individual transport measurements and individual transport modeling for distinct SEIs.

In the framework of our model, the current density ratio shown in Figure 4(b) can be expressed as:

(26) $$\frac{j_{{Fc}^{+}}}{j_{carbonate}}=\frac{c_{eon}^{x=0}\;D_{eon}+\epsilon \;c_{{Fc}^{+}}^{bulk}\;D_{{Fc}^{+}}}{c_{eon}^{x=0}\;D_{eon}},$$

Based on this equation, an Onsager transport coefficient of the Fc^+^ ions in the SEI can be calculated using the Nernst‐Einstein relation:

(27) $$\sigma_{{Fc}^{+}}=\frac{F^{2}}{R\;T}\epsilon \;c_{{Fc}^{+}}^{bulk}\;D_{{Fc}^{+}}=\sigma_{eon}\left(\frac{j_{{Fc}^{+}}}{j_{carbonate}}-1\right).$$

As seen from Eq. (27), reliable values for *σ*_FC_^+^ can be obtained, if the current density ratio deviates significantly from unity. As seen from Figure 4(b), this is the case at formation potentials of 0.6 V and 0.8 V and at SEI formation times up to about 16 hours. We note that the Onsager transport coefficient of the organocarbonate molecules *σ*_carbonate_ is about 10^3^ higher than *σ*_FC_^+^, simply due to approximately 10^3^ times higher concentration of the organocarbonate molecules.

**Figure 12:**
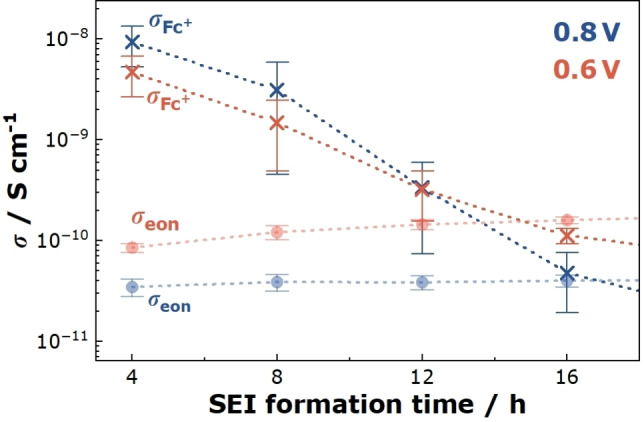
Onsager transport coefficient of the Fc^+^ ions, $\sigma_{{Fc}^{+}}$ , and the Onsager transport coefficient of the electrons, $\sigma_{eon}$ , plotted versus the SEI formation time at 0.6 and 0.8 V vs. Li^+^/Li, respectively.

In Figure 12, we plot *σ*_FC_^+^ and *σ*_eon_ versus the SEI formation time at 0.6 V and 0.8 V, respectively. After 4 hours of SEI formation, *σ*_FC_^+^ is in the range of 10^−8^ S cm^−1^, i. e. 1.5 to 2.5 orders of magnitude higher than *σ*_eon_. After 16 hours of SEI formation time, *σ*_FC_^+^ has dropped to values close to *σ*_eon_. At longer SEI formation times, *σ*_FC_^+^ drops below *σ*_eon_, resulting in an electron transport limitation for both redox molecule and organocarbonate molecule reduction. At an SEI formation potential of 0.1 V, *σ*_FC_^+^<*σ*_eon_ is already valid at short SEI formation times, indicating a very low porosity of the SEI.

## Conclusions

The ratio of the redox molecule reduction current density to the organocarbonate reduction current density at SEI‐covered glassy carbon electrodes was measured by means of a generator‐collector experiment and was found to decrease with increasing SEI formation time and with decreasing SEI formation potential. The experimental results were compared to a transport and reaction model accounting for molecule‐electron reactions inside the SEI as well as in the double layer at the SEI | bulk electrolyte interface. It was shown that this model can be mapped onto a Z‐type transmission‐line model with P−Q conditions. The model predicts four distinct transport and reaction regimes depending on the kinetic rate constant for the molecule‐electron reactions. A comparison of the experimental results with this model gives strong indication that the organocarbonate reduction takes place mainly in the double layer at the SEI | bulk electrolyte interface and is governed by electron transport across the SEI. In contrast, the redox molecule transport and reaction mechanism depends on the porosity of the SEI and thus on the SEI formation time. When the porosity is still relatively high at short formation times, redox molecules diffuse across the SEI and are reduced close to the electrode | SEI interface. With decreasing porosity, the reaction shifts towards the SEI | bulk electrolyte interface. For SEIs formed at lower potentials, this shift takes place at shorter SEI formation times. Owing to the distinct transport and reaction mechanisms of organocarbonate molecules and of redox molecules, good estimates can be obtained for the Onsager transport coefficients of electrons and molecules.

We note that in a first step taken here, the combined experimental and modeling approach was applied to a model‐type SEI formed on a planar electrode in the absence of lithium intercalation. However, the approach should also be applicable to SEIs formed on composite graphite electrodes used in lithium‐ion batteries. Furthermore, the approach can also be used for characterizing interphases in other fields of electrochemistry, such as electrocatalysis and corrosion protection. This should contribute to a deeper understanding of transport and reaction mechanisms in electrocatalytic layers as well as in corrosion passivation layers.

## Supporting Information

Comprehensive supplementary material is provided, encompassing the determination of the distance between generator electrode and collector electrode, the analysis of impedance spectra in the elastance plane, additional chronoamperometric data, simulation of the generator‐collector experiment, and analytical solutions of Z‐type TLM with P−Q condition in different transport and reaction regimes. The authors have given additional references within the Supporting Information.[^25^, ^36^, ^44^, ^53^, ^54^, ^55^, ^56^]

## Conflict of Interests

The authors declare no conflict of interest.

1

## Supporting information

As a service to our authors and readers, this journal provides supporting information supplied by the authors. Such materials are peer reviewed and may be re‐organized for online delivery, but are not copy‐edited or typeset. Technical support issues arising from supporting information (other than missing files) should be addressed to the authors.

Supporting Information

## Data Availability

The data that support the findings of this study are available in the supplementary material of this article.
