# Supplementary material for: Transport and Reaction of Electrons and Molecules in Solid Electrolyte Interphases formed at Different Electrode Potentials: A Combined Experimental and Modeling Approach
Source: ChemSusChem. 2025 Apr 2;18(10):e202402468. doi: 10.1002/cssc.202402468 (PMC12094159; doi:10.1002/cssc.202402468)
Supplement: Supplementary file 1 — Supporting Information [file CSSC-18-e202402468-s001.pdf]

ChemSusChem

Supporting Information

Transport and Reaction of Electrons and Molecules in Solid Electrolyte Interphases formed at Different Electrode Potentials: A Combined Experimental and Modeling Approach

Falk Thorsten Krauss, Annalena Duncker, and Bernhard Roling*

Supporting Information for

Transport and Reaction of Electrons and Molecules in Solid Electrolyte Interphases formed at Different Potentials: A Combined Experimental and Modeling Approach

Falk Thorsten Krauss‡, Annalena Duncker‡, and Bernhard Roling*

Department of Chemistry, Philipps-Universität Marburg, Hans-Meerwein-Straße 4, 35032 Marburg, Germany

‡These authors contributed equally.

*Email: roling@staff.uni-marburg.de

1. Determination of the distance between generator electrode and collector electrode

Impedance spectra of the liquids m-xylene and n-dodecane between the generator electrode and the collector electrode (two-electrode setup) were taken. The impedance spectra were converted into capacitance spectra via:

$$\hat{C}(\omega) = \frac{1}{i\omega\hat{Z}(\omega)} \tag{S1}$$

Figure S1 show a representative plot of the real part of the capacitance of m-xylene, $C'(\omega)$, in a frequency range from 10^6 Hz to 10^3 Hz. A linear fit in the bulk plateau region in the range of 10^5 Hz to 10^4 Hz yields a capacitance of (6.03 ± 0.05) pF for m-xylene and (5.26 ± 0.05) pF for n-dodecane, respectively.

In a parallel-plate setup with two electrode of different diameter, the capacitance is given by:[1]

$$C = \frac{\varepsilon_0 \cdot \varepsilon_r \cdot 4\pi D^2}{16 \cdot w} + \varepsilon_0 \cdot \varepsilon_r \cdot D \cdot \left(\ln\left(\frac{4\pi D}{w}\right) - 3\right). \tag{S2}$$

Here, $D = 4$ mm is the diameter of the smaller generator electrode, while w is the distance between the two electrodes. $\varepsilon_0 = 8.854 \cdot 10^{-12}\ \frac{\mathrm{As}}{\mathrm{Vm}}$ denotes the permittivity of free space. Eq. (S2) is valid, if the difference of the electrode diameters (here 2 mm) is at least three times larger than w, which is the case here.

With the relative permittivity of m-xylene, $\varepsilon_r = 2.357$,[2] and of n-dodecane, $\varepsilon_r = 2.020$,[3] an electrode distance of $w = 46.0\ \mu m$ (m-xylene) and $w = 45.0\ \mu m$ (n-dodecane) was obtained. Averaging these two values yields $(45.5 \pm 0.4)\ \mu m$.

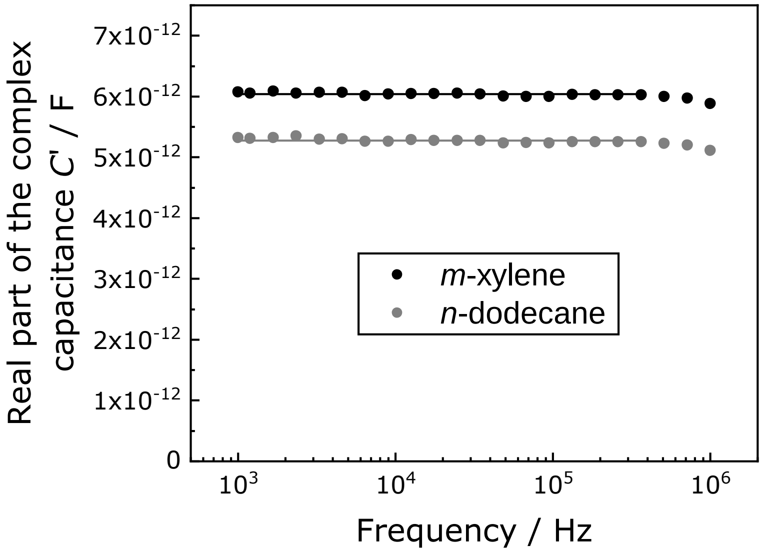

Figure S1. *Real part of the complex capacitance of m-xylene and n-dodecane in a frequency range from* $10^6\ Hz$ *to* 10^3 *Hz. The solid lines show a linear fit in the bulk plateau regime.*

2. Analysis of impedance spectra in the elastance plane

The frequency-dependent elastance $\hat{S}(\omega)$ was calculated from the frequency-dependent impedance $\hat{Z}(\omega)$ via:

$$\hat{S}(\omega) = \mathrm{i}\omega\hat{Z}(\omega) = S' + \mathrm{i}S'' \qquad \text{(S3)}$$

Here, i is the imaginary unit. The high-frequency branch of the spectra originating from the electrolyte resistance was fitted by a line, and the linear fit was extrapolated to the S' axis, see Figure S2. As shown in Ref. [4], the S' axis intercept is given by $\frac{1}{C_{SEI}} + \frac{1}{C_{DL}}$, which is virtually identical to $\frac{1}{C_{SEI}}$, since the double layer capacitance C_{DL} is much higher than the SEI capacitance C_{SEI}. Since the porosity of the inner SEI is low already at short SEI formation times, it is likely that the SEI composition is virtually constant and that also the relative permittivity of the SEI is virtually

constant, even if the inner layer of the SEI is heterogeneous. Assuming that the relative permittivity of the SEI is $\varepsilon_r = 10$, the SEI thickness was calculated as:

$$d_{SEI} = \frac{\varepsilon_0 \varepsilon_r A}{C_{SEI}} \quad \text{(S4)}$$

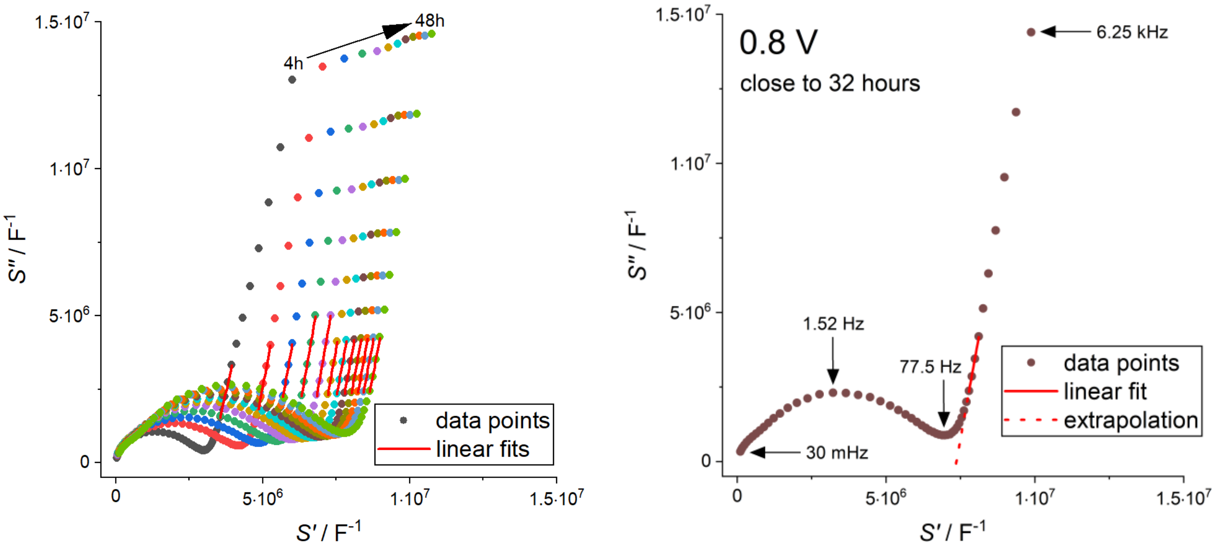

Figure S2. *Impedance spectra in the elastance plane during SEI formation at 0.8 V vs. Li$^+$/Li. Left: Impedance spectra at different SEI formation time including linear fits of the high-frequency branch. Right: Representative impedance spectrum obtained close to 32 hours of SEI formation time including linear fit of the high-frequency branch.*

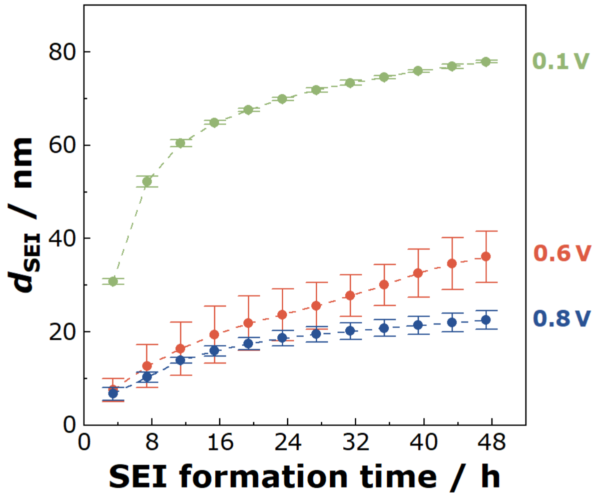

Figure S3. *Temporal evolution of the SEI thickness obtained from the elastance spectra shown in Fig. S2.*

3. Additional chronoamperometric data

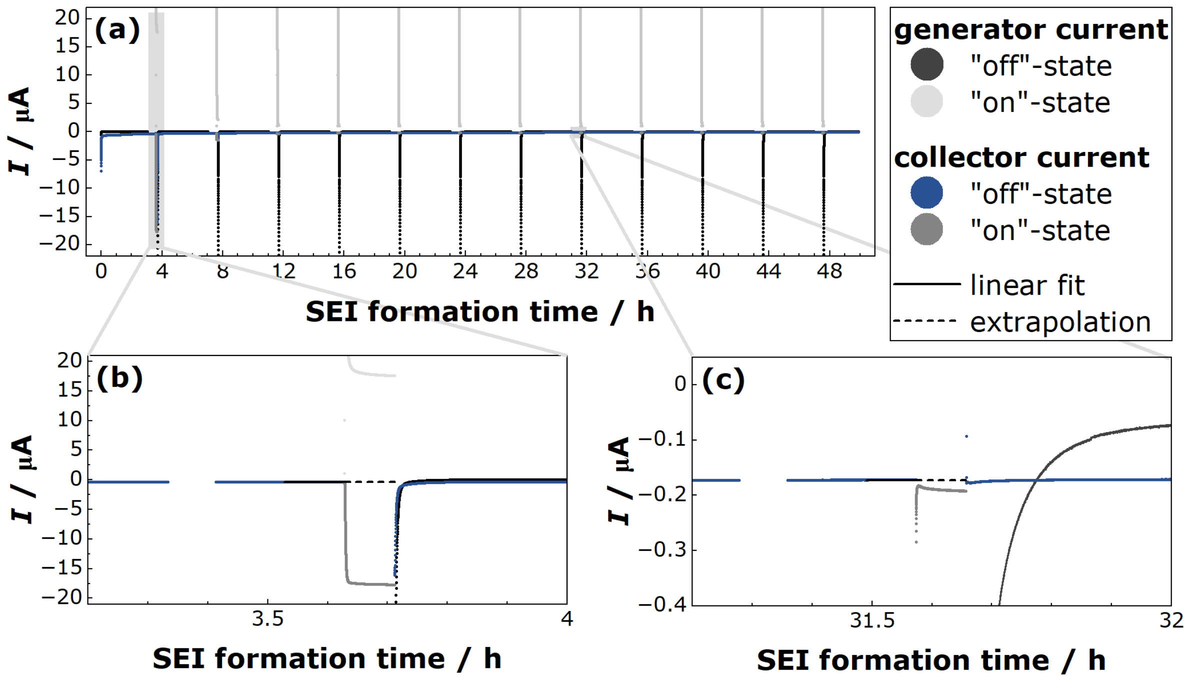

Figure S4. *Measured currents during SEI formation at 0.8 V vs.* Li^{+}/Li *and during the generator-collector experiments.*

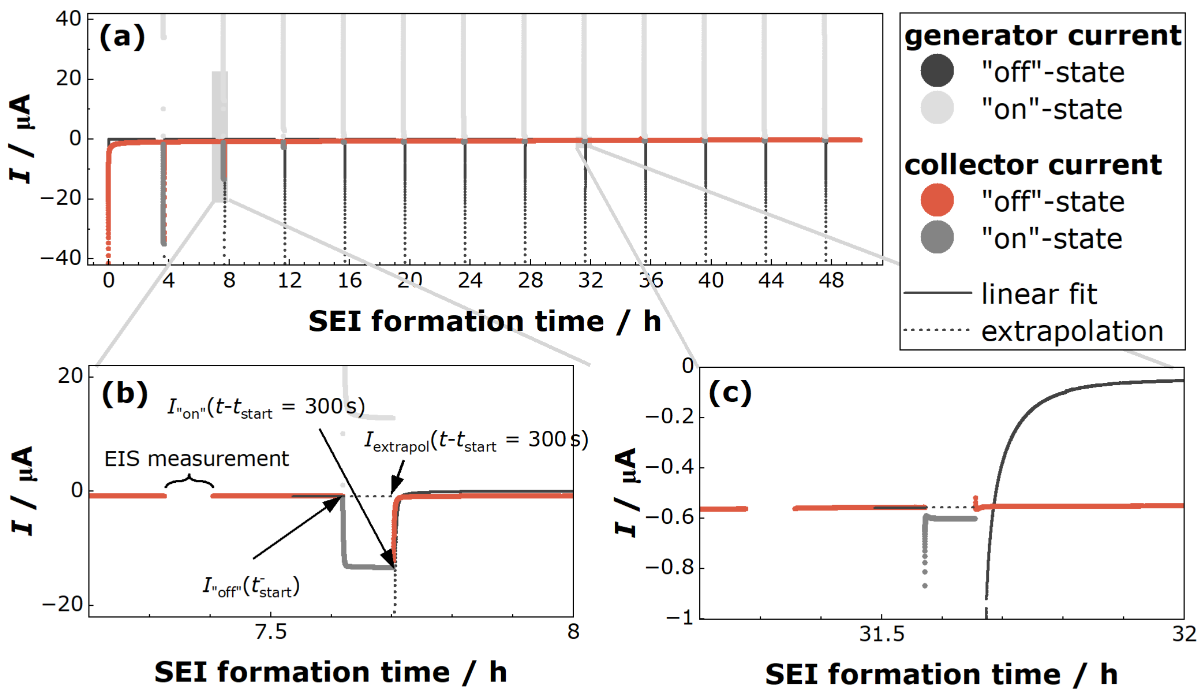

Figure S5. *Measured currents during SEI formation at 0.6 V vs. Li⁺/Li and during the generator-collector experiments.*

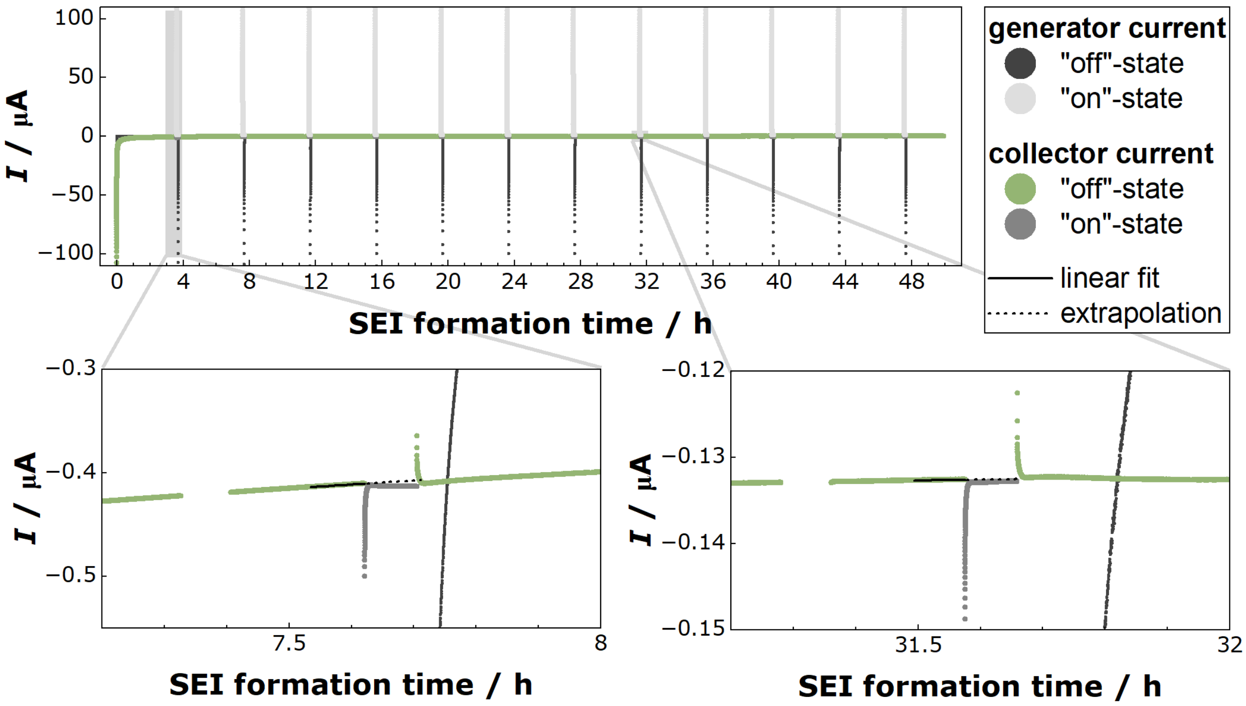

Figure S6. *Measured currents during SEI formation at 0.1 V vs. Li⁺/Li and during the generator-collector experiments.*

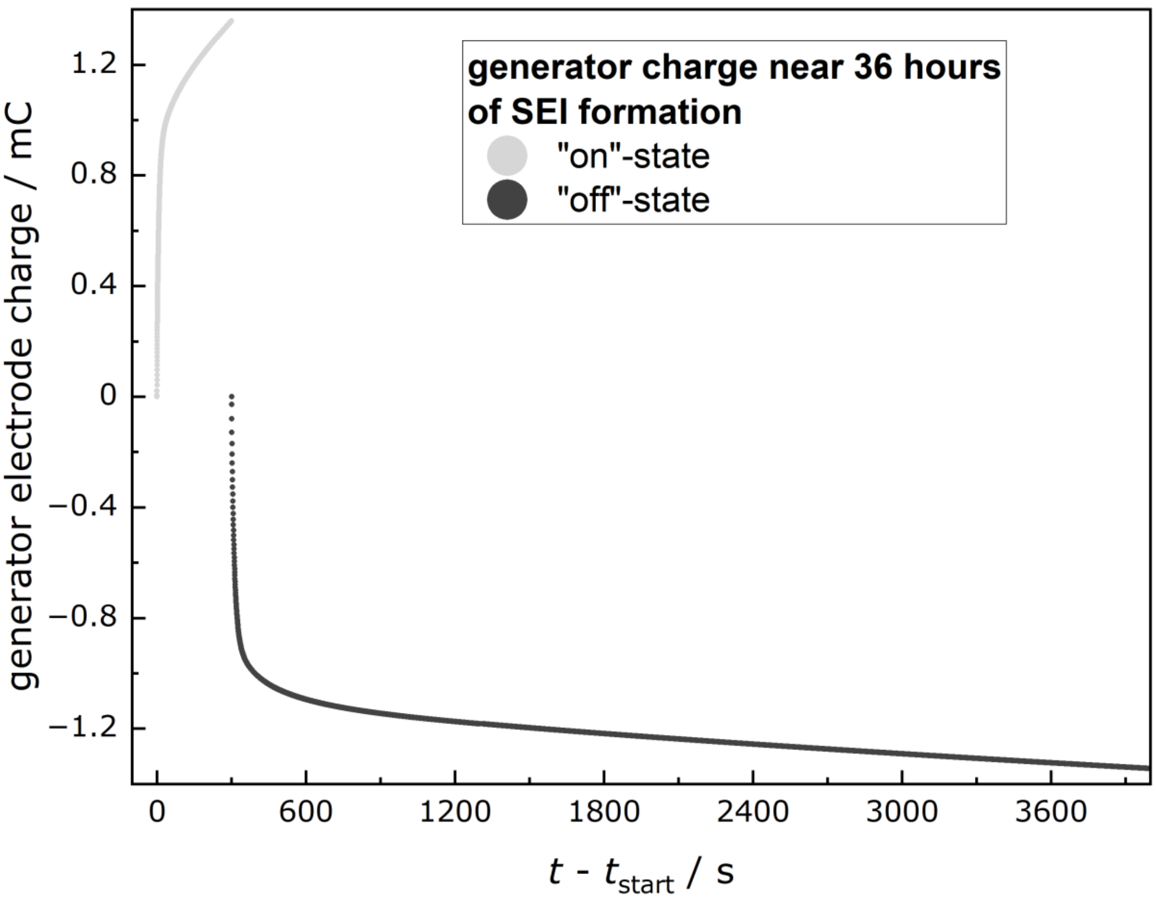

Figure S7. *Integrated charge curves for a representative generator-collector experiment close to 32 hours of SEI formation at 0.8 V vs. Li^+/Li. Grey data points: Integrated charge at the generator electrode during the "on"-state (t-t_{start} = 0 – 300 s) due to Fc oxidation. Black data points: Integrated charge during the "off"-state (t-t_{start} > 300 s) due to Fc^+ ion reduction. About one hour after the "on" → "off" switching, the reductive charge at the generator electrode is virtually identical to the oxidative charge in the "on"-state*.

4. Simulation of the generator-collector experiment

The simulations were performed by means of the *Electroanalysis* module of COMSOL Multiphysics 5.0 (COMSOL Inc.). A generator-collector experiment on a $\mathrm{Fc^+/Fc}$ redox couple

$$\mathrm{Fc} \leftrightarrows \mathrm{Fc^+} + \mathrm{e^-}$$

was simulated using the same length scales as in the experimental setup. The transport of Fc and Fc^+ in solution was simulated under supporting electrolyte conditions using Fick's second law:

$$\frac{\mathrm{d}c_i}{\mathrm{d}t} = D\,\nabla^2 c_i \tag{S5}$$

with $i = \mathrm{Fc^+}, \mathrm{Fc}$. The diffusion coefficients of both species were assumed to be identical, $D_{Fc} = D_{Fc^+} = D$. Initial concentration of the species are $c_{\mathrm{Fc^+}}(t = 0) = 10\ \mathrm{mM}$ and $c_{\mathrm{Fc}}(t = 0) = 0$, respectively. Fick's second law was solved numerically in a cylindrical coordinate system with r denoting the radial distance and z denoting the axial coordinate.

The rate equation (S6) was utilized to compute the time-dependent current density $j_{\mathrm{generator}}(t)$ due to the heterogenous charge transfer reaction at the generator electrode:

$$j_{\mathrm{generator}}(t) = F\,k_{\mathrm{ox}}\,c_{\mathrm{Fc}}(z = w, t) \tag{S6}$$

with k_{ox} and F denoting the rate constant of the ferrocenium oxidation reaction and the Faraday constant, respectively. The rate equation (S7) was utilized to compute the time-dependent current density $j_{\mathrm{collector}}(t)$ due to the charge transfer reaction at the SEI-covered collector electrode:

$$j_{\mathrm{collector}}(t) = -\,F\,k_{\mathrm{eff}}\,c_{\mathrm{Fc^+}}(z = 0, t). \tag{S7}$$

Here k_{eff} is an effective rate constant of the ferrocenium ion reduction reaction at the SEI-covered collector electrode. At the cell walls, the insulating boundary conditions:

$$\nabla c_i = 0 \tag{S8}$$

hold with $i = \mathrm{Fc^+}, \mathrm{Fc}$.

All boundary conditions of the simulation are depicted in Figure S8, and the simulation parameters are listed in Table S1. The computational mesh of the model is shown in Figure S9.

In Figure S10, spatial images of the concentration of ferrocenium ions during the "on" state at to $t - t_{\mathrm{start}} = 300\ \mathrm{s}$ are depicted for three different cases: Case A with $k_{\mathrm{eff}} \gg \frac{D_{\mathrm{Fc}^+}}{w}$ applies for an SEI-free bare collector electrode exhibiting an effective rate constant of $k_{\mathrm{eff}} = 0.42\ \mathrm{m\ s^{-1}}$, see Figure S10(a); Case B with $k_{\mathrm{eff}} \approx \frac{D_{\mathrm{Fc}^+}}{w}$ applies for a collector electrode covered with a poorly passivating SEI exhibiting $k_{\mathrm{eff}} = 4.2 \cdot 10^{-6}\ \mathrm{m\ s^{-1}}$, see Figure S10(b); Case C with $k_{\mathrm{eff}} \gg \frac{D_{\mathrm{Fc}^+}}{w}$ applies for a collector electrode covered with a highly passivating SEI exhibiting $k_{\mathrm{eff}} = 4.2 \cdot 10^{-11}\ \mathrm{m\ s^{-1}}$, see Figure S10(c).

The simulations results in Figure S10 show that for all cases A, B and C, the Fc^+ ion reduction reaction at the collector electrode takes place, to a good approximation, on a fraction of the collector electrode surface area, which is identical to the generator electrode surface area.

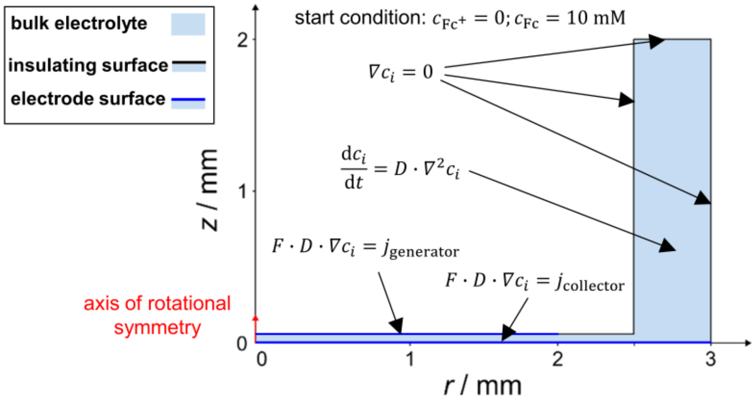

Figure S8. *Illustration of the simulations in cylinder coordinates with axis of rotational symmetry at $r = 0$. $j_{\mathrm{generator}}$ and $j_{\mathrm{collector}}$ denotes the current density according to Eqs. (S6) and (S7), respectively.*

Table S1. *Simulation parameters.*

Parameter	Value	Description
r		radial distance
z		axial coordinate
w	$45.5 \cdot 10^{-6}$ m	distance between generator and collector electrode
$r_{\text{generator}}$	$2 \cdot 10^{-3}$ m	radius of the generator electrode
$r_{\text{collector}}$	$3 \cdot 10^{-3}$ m	radius of the collector electrode
$c_{\text{Fc}}^{\text{bulk}}$	10 mol m^{-3}	concentration of Fc in the bulk solution
D	$3.76 \cdot 10^{-10}$ m^2 s^{-1}	diffusion coefficient of both redox species (assuming: $D = D_{Fc^+} = D_{Fc}$)[6]
k_{ox}	0.016 m s^{-1}	heterogenous rate constant of the Fc oxidation reaction at the generator electrode
k_{eff}	*variable parameter*	effective rate constant of the Fc^+ ion reduction reaction at the SEI-covered collector electrode
F	96485.332 C mol^{-1}	Faraday constant

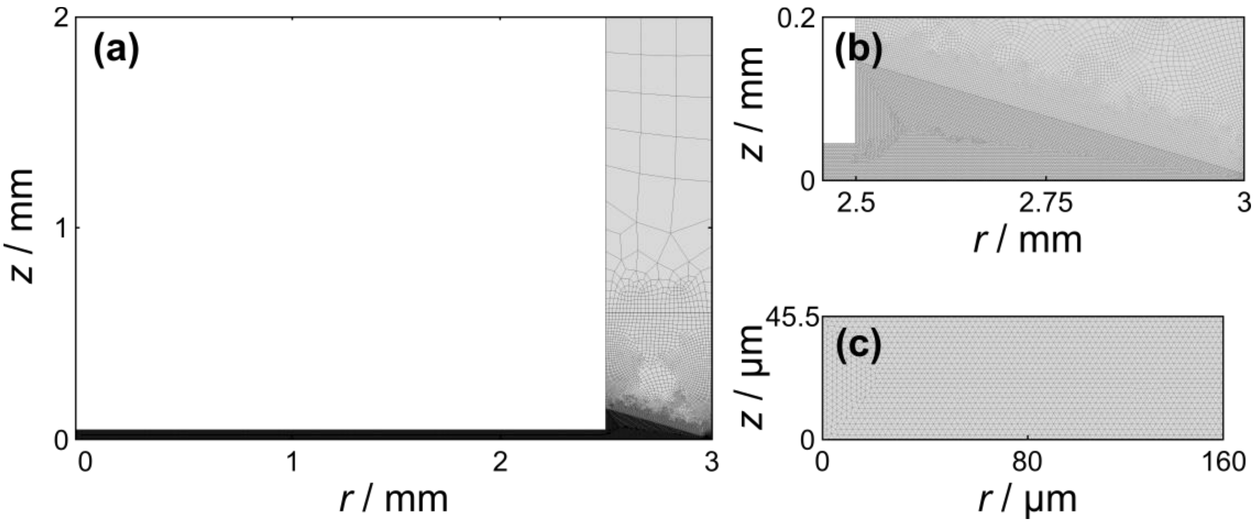

Figure S9. *Computational mesh of the simulation box. (a) Complete simulation box; (b) transition regime between the fine mesh near the electrode surfaces and the coarse mesh in the electrolyte reservoir; (c) fine mesh between the electrode surfaces.*

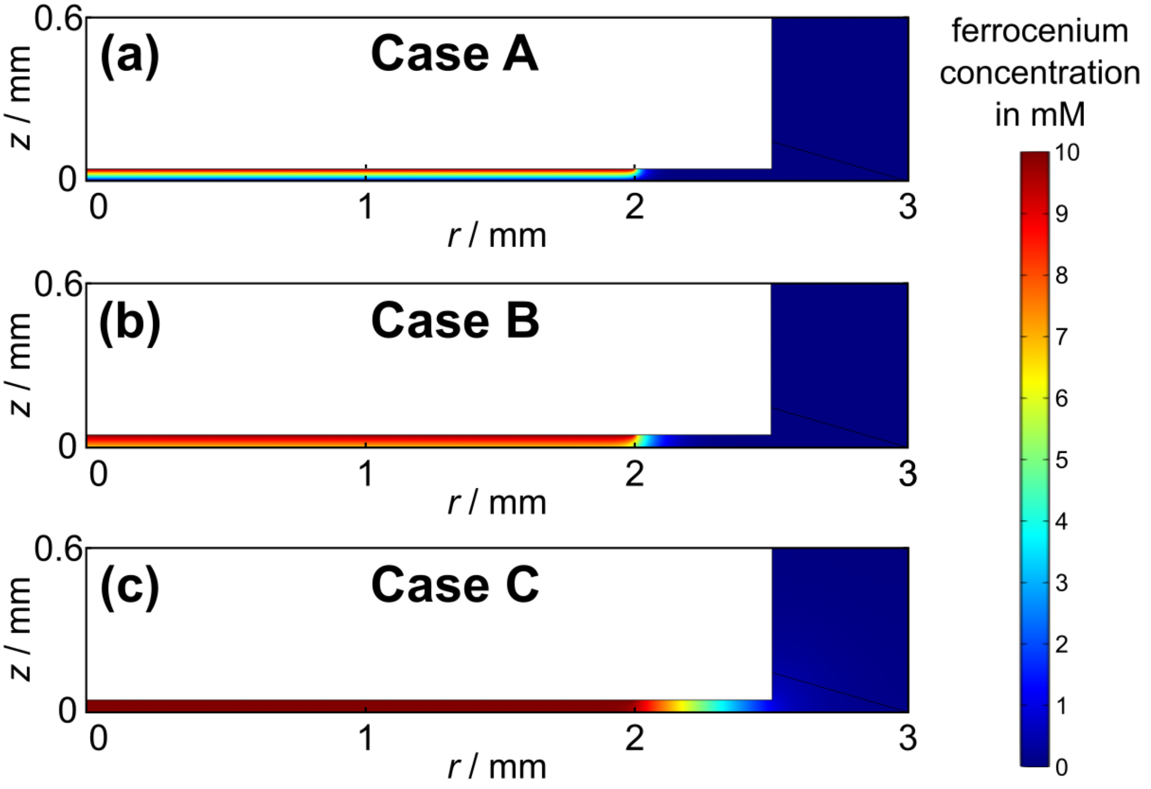

Figure S10. *Spatial images of the concentration of Fc*$^{+}$ *ions during the "on"-state at* $t - t_{start} = 300\,\mathrm{s}$ *for three different cases: Case A with* $k_{\mathrm{eff}} \gg \frac{D_{\mathrm{Fc}^+}}{w}$ *(bare collector electrode with no SEI;* $k_{\mathrm{eff}} = 0.42\ \mathrm{m\ s^{-1}}$*); Case B with* $k_{\mathrm{eff}} \approx \frac{D_{Fc^+}}{w}$ *(collector electrode covered with a poorly passivating SEI;* $k_{\mathrm{eff}} = 4.2 \cdot 10^{-6}\ \mathrm{m\ s^{-1}}$*); Case C with* $k_{\mathrm{eff}} \gg \frac{D_{\mathrm{Fc}^+}}{w}$ *(collector electrode covered with a highly passivating SEI;* $k_{\mathrm{eff}} = 4.2 \cdot 10^{-11}\ \mathrm{m\ s^{-1}}$*).*

5. Analytical solutions of the Z-type Transmission-Line Model (TLM) with P-Q conditions in different transport and reaction regimes

In the framework of the Z-type TLM with P-Q conditions, the inverse dimensionless current density is given by:[7]

$$\frac{1}{\tilde{J}_i} = \frac{\tilde{r}_i\,\tilde{r}_{\text{eon}}}{\tilde{r}_i + \tilde{r}_{\text{eon}}}\,\tilde{d}_{\text{SEI}} + \frac{\sqrt{\tilde{r}_{\text{CT}}^{\text{SEI}}}}{(\tilde{r}_i + \tilde{r}_{\text{eon}})^{3/2}}\;\frac{\left(\tilde{r}_i{}^2 + \tilde{r}_{\text{eon}}{}^2\right)\cosh\beta + 2\,\tilde{r}_i\,\tilde{r}_{\text{eon}} + \left(\frac{\tilde{r}_i{}^2}{\tilde{R}_{\text{CT}}^{\text{SEI}}} + \frac{\tilde{r}_{\text{eon}}{}^2}{\tilde{R}_{\text{CT}}^{\text{int}}}\right)\cdot\sqrt{\tilde{r}_{\text{CT}}^{\text{SEI}}\,(\tilde{r}_i + \tilde{r}_{\text{eon}})}\cdot\sinh\beta}{\left(1 + \frac{\tilde{r}_{\text{CT}}^{\text{SEI}}\,(\tilde{r}_i + \tilde{r}_{\text{eon}})}{\tilde{R}_{\text{CT}}^{\text{SEI}}\,\tilde{R}_{\text{CT}}^{\text{int}}}\right)\sinh\beta + \left(\frac{1}{\tilde{R}_{\text{CT}}^{\text{SEI}}} + \frac{1}{\tilde{R}_{\text{CT}}^{\text{int}}}\right)\cdot\sqrt{\tilde{r}_{\text{CT}}^{\text{SEI}}\,(\tilde{r}_i + \tilde{r}_{\text{eon}})}\,\cosh\beta} \tag{S9}$$

with

$$\beta = \sqrt{\frac{\tilde{r}_i + \tilde{r}_{\text{eon}}}{\tilde{r}_{\text{CT}}^{\text{SEI}}}}\cdot\tilde{d}_{\text{SEI}} \tag{S10}$$

The model parameters were chosen in a way that two conditions are fulfilled: First, the transport resistance of the molecules is much lower than the transport resistance of the electrons: $\tilde{r}_{\text{i}}\,\tilde{d}_{\text{SEI}} \ll \tilde{r}_{\text{eon}}\,\tilde{d}_{\text{SEI}}$. Second, the porosity of the SEI is low, so that the charge transfer resistance in the SEI is much higher than the charge transfer resistance at the SEI | bulk electrolyte interface: $\tilde{r}_{\text{CT}}^{\text{SEI}}/\tilde{d}_{\text{SEI}} \gg \tilde{R}_{\text{CT}}^{\text{int}}$. The parameters chosen for Fig. 6 were $\varepsilon = 10^{-7}$, $\tilde{c}_i^{\text{bulk}} = 1$, $\tilde{D} = 1$, $\tilde{D}_{\text{eon}} = 0.1$, $\tilde{c}_{\text{eon}}^{\tilde{x}=0} = 10^{-8}$ and $\tilde{d}_{\text{SEI}} = 100$, leading to the following dimensionless resistance values:

$$\tilde{r}_i\,\tilde{d}_{\text{SEI}} = \frac{\tilde{d}_{\text{SEI}}}{\varepsilon\,\tilde{c}_i^{\text{bulk}}\,\tilde{D}} = 10^9 \tag{S11}$$

$$\tilde{r}_{\text{eon}}\,\tilde{d}_{\text{SEI}} = \frac{\tilde{d}_{\text{SEI}}}{\tilde{c}_{\text{eon}}^{\tilde{x}=0}\,\tilde{D}_{\text{eon}}} = 10^{11} \tag{S12}$$

$$\tilde{r}_{\text{CT}}^{\text{SEI}}/\,\tilde{d}_{\text{SEI}} = \frac{1}{\tilde{k}_i\,\tilde{c}_{\text{eon}}^{\tilde{x}=0}\,\varepsilon\,\tilde{c}_i^{\text{bulk}}\,\tilde{d}_{\text{SEI}}} = 10^{13}/\tilde{k}_i \tag{S13}$$

$$\tilde{R}_{\text{CT}}^{\text{SEI}} = \frac{1}{\tilde{k}_i\,\tilde{c}_{\text{eon}}^{\tilde{x}=0}\,\varepsilon\,\tilde{c}_i^{\text{bulk}}\,\tilde{d}_{\text{DL}}} = 10^{15}/\tilde{k}_i \tag{S14}$$

$$\tilde{R}_{\text{CT}}^{\text{int}} = \frac{1}{\tilde{k}_i\,\tilde{c}_{\text{eon}}^{\tilde{x}=0}\,\tilde{c}_i^{\text{bulk}}\tilde{d}_{\text{DL}}} = 10^{8}/\tilde{k}_i \tag{S15}$$

Depending on the dimensionless rate constant $\tilde{k}_{\text{i}}$, four distinct transport and reaction regimes can be distinguished, and simplified analytical expressions can be obtained in these regimes.

Regime (i)

At very low rate constants $\tilde{k}_i$, the charge-transfer resistance inside the SEI, $\tilde{r}_{\mathrm{CT}}^{\mathrm{SEI}}/\tilde{d}_{\mathrm{SEI}}$, is very large and thus the parameter β goes to zero. Consequently, we use the approximations:

$$\lim_{\beta \to 0} \sinh(\beta) = 0 \tag{S16}$$

and

$$\lim_{\beta \to 0} \cosh(\beta) = 1 \tag{S17}$$

In this case, Eq. (S9) simplifies to:

$$\frac{1}{\tilde{j}_i} = \frac{\tilde{r}_i\, \tilde{r}_{\mathrm{eon}}}{r_i + \tilde{r}_{\mathrm{eon}}}\, \tilde{d}_{\mathrm{SEI}} + \tilde{R}_{\mathrm{CT}}^{\mathrm{int}}. \tag{S18}$$

Since $\tilde{R}_{\mathrm{CT}}^{\mathrm{int}}$ is very large in this regime, the term $\frac{\tilde{r}_i\, \tilde{r}_{\mathrm{eon}}}{r_i + \tilde{r}_{\mathrm{eon}}}\, \tilde{d}_{\mathrm{SEI}}$ can be neglected, resulting in:

$$\tilde{j}_i = \frac{1}{\tilde{R}_{\mathrm{CT}}^{\mathrm{int}}} = \tilde{k}_i\, \tilde{c}_i^{\mathrm{bulk}}\, \tilde{c}_{\mathrm{eon}}^{x=0}\, \tilde{d}_{\mathrm{DL}}. \tag{S19}$$

Regime (ii)

At higher rate constants in the range of $\tilde{k}_i = 1$, we use the approximations $\sinh(\beta) \approx \beta$ and $\cosh(\beta) \approx 1$, which, together with $\tilde{r}_{\mathrm{i}}\, \tilde{d}_{\mathrm{SEI}} \ll \tilde{r}_{\mathrm{eon}}\, \tilde{d}_{\mathrm{SEI}}$, result in:

$$\frac{1}{\tilde{j}_i} = \tilde{r}_i\, \tilde{d}_{\mathrm{SEI}} + \frac{\sqrt{\tilde{r}_{\mathrm{CT}}^{\mathrm{SEI}}}}{(\tilde{r}_{\mathrm{eon}})^{3/2}}\; \frac{\tilde{r}_{\mathrm{eon}}{}^2 + \left(\frac{\tilde{r}_i{}^2}{\tilde{R}_{\mathrm{CT}}^{\mathrm{SEI}}} + \frac{\tilde{r}_{\mathrm{eon}}{}^2}{\tilde{R}_{\mathrm{CT}}^{\mathrm{int}}}\right) \cdot \sqrt{\tilde{r}_{\mathrm{CT}}^{\mathrm{SEI}}\, \tilde{r}_{\mathrm{eon}}} \cdot \beta}{\left(1 + \frac{\tilde{r}_{\mathrm{CT}}^{\mathrm{SEI}}\, \tilde{r}_{\mathrm{eon}}}{\tilde{R}_{\mathrm{CT}}^{\mathrm{SEI}}\, \tilde{R}_{\mathrm{CT}}^{\mathrm{int}}}\right) \cdot \beta + \left(\frac{1}{\tilde{R}_{\mathrm{CT}}^{\mathrm{SEI}}} + \frac{1}{\tilde{R}_{\mathrm{CT}}^{\mathrm{int}}}\right) \cdot \sqrt{\tilde{r}_{\mathrm{CT}}^{\mathrm{SEI}}\, \tilde{r}_{\mathrm{eon}}}}. \tag{S20}$$

With $\beta = \sqrt{\frac{\tilde{r}_i + \tilde{r}_{\mathrm{eon}}}{\tilde{r}_{\mathrm{CT}}^{\mathrm{SEI}}}} \cdot \tilde{d}_{\mathrm{SEI}} \approx \sqrt{\frac{\tilde{r}_{\mathrm{eon}}}{\tilde{r}_{\mathrm{CT}}^{\mathrm{SEI}}}} \cdot \tilde{d}_{\mathrm{SEI}}$, Eq. (S20) further simplifies to:

$$\frac{1}{\tilde{j}_i} = \tilde{r}_i\, \tilde{d}_{\mathrm{SEI}} + \frac{\tilde{R}_{\mathrm{CT}}^{\mathrm{int}} + \frac{\tilde{R}_{\mathrm{CT}}^{\mathrm{int}} \cdot \tilde{r}_i{}^2}{\tilde{R}_{\mathrm{CT}}^{\mathrm{SEI}} \cdot \tilde{r}_{\mathrm{eon}}} \cdot \tilde{d}_{\mathrm{SEI}} + \tilde{r}_{\mathrm{eon}}\, \tilde{d}_{\mathrm{SEI}}}{\frac{\tilde{R}_{\mathrm{CT}}^{\mathrm{int}}}{\tilde{r}_{\mathrm{CT}}^{\mathrm{SEI}}} \cdot \tilde{d}_{\mathrm{SEI}} + \frac{\tilde{r}_{\mathrm{eon}}}{\tilde{R}_{\mathrm{CT}}^{\mathrm{SEI}}} \cdot \tilde{d}_{\mathrm{SEI}} + \frac{\tilde{R}_{\mathrm{CT}}^{\mathrm{int}}}{\tilde{R}_{\mathrm{CT}}^{\mathrm{SEI}}} + 1}. \tag{S21}$$

For $\tilde{k}_i = 1$, the terms $\frac{\tilde{R}_{\mathrm{CT}}^{\mathrm{int}}}{\tilde{r}_{\mathrm{CT}}^{\mathrm{SEI}}} \tilde{d}_{\mathrm{SEI}} = 10^{-5}$, $\frac{\tilde{r}_{\mathrm{eon}}}{\tilde{R}_{\mathrm{CT}}^{\mathrm{SEI}}} \tilde{d}_{\mathrm{SEI}} = 10^{-4}$ and $\frac{\tilde{R}_{\mathrm{CT}}^{\mathrm{int}}}{\tilde{R}_{\mathrm{CT}}^{\mathrm{SEI}}} = 10^{-7}$ in the denominator are all negligible, leading to:

$$\frac{1}{\tilde{J}_i} = \tilde{r}_i\, \tilde{d}_{\mathrm{SEI}} + \tilde{R}_{\mathrm{CT}}^{\mathrm{int}} + \frac{\tilde{R}_{\mathrm{CT}}^{\mathrm{int}} \cdot \tilde{r}_i^{\,2}}{\tilde{R}_{\mathrm{CT}}^{\mathrm{SEI}} \cdot \tilde{r}_{\mathrm{eon}}} \cdot \tilde{d}_{\mathrm{SEI}} + \tilde{r}_{\mathrm{eon}}\, \tilde{d}_{\mathrm{SEI}}. \tag{S22}$$

Since the terms $\tilde{r}_i\, \tilde{d}_{\mathrm{SEI}} = 10^9$, $\tilde{R}_{\mathrm{CT}}^{\mathrm{int}} = 10^8$ and $\frac{\tilde{R}_{\mathrm{CT}}^{\mathrm{int}}\, \tilde{r}_i^{\,2}}{\tilde{R}_{\mathrm{CT}}^{\mathrm{SEI}}\, \tilde{r}_{\mathrm{eon}}} \tilde{d}_{\mathrm{SEI}} = 1$ are all much lower than $\tilde{r}_{\mathrm{eon}}\, \tilde{d}_{\mathrm{SEI}} = 10^{11}$, we obtain finally:

$$\frac{1}{\tilde{J}_i} = \tilde{r}_{\mathrm{eon}}\, \tilde{d}_{\mathrm{SEI}}. \tag{S23}$$

and

$$\tilde{J}_i = \frac{1}{\tilde{r}_{\mathrm{eon}}\, \tilde{d}_{\mathrm{SEI}}} = \frac{\tilde{D}_{\mathrm{eon}}\, \tilde{c}_{\mathrm{eon}}^{x=0}}{\tilde{d}_{\mathrm{SEI}}}. \tag{S24}$$

Regime (iii)

With further increasing rate constant $\tilde{k}_i$, the parameter β reaches very large values. In this case, the sinh and cosh functions can be approximated by

$$\lim_{\beta\to\infty} \sinh(\beta) = \lim_{\beta\to\infty} \cosh(\beta) = \frac{e^{\beta}}{2}. \tag{S25}$$

Since $\tilde{r}_i\, \tilde{r}_{\mathrm{eon}} \ll \tilde{r}_{\mathrm{eon}}^{\,2}$ holds, the term $2\, \tilde{r}_i\, \tilde{r}_{\mathrm{eon}}$ in the numerator of Eq. (S9) can be neglected, and Eq. (S9) simplifies to:

$$\frac{1}{\tilde{J}_i} = \tilde{r}_i\, \tilde{d}_{\mathrm{SEI}} + \frac{\sqrt{\tilde{r}_{\mathrm{CT}}^{\mathrm{SEI}}}}{(\tilde{r}_{\mathrm{eon}})^{3/2}}\; \frac{\tilde{r}_{\mathrm{eon}}^{\,2} + \left(\frac{\tilde{r}_i^{\,2}}{\tilde{R}_{\mathrm{CT}}^{\mathrm{SEI}}} + \frac{\tilde{r}_{\mathrm{eon}}^{\,2}}{\tilde{R}_{\mathrm{CT}}^{\mathrm{int}}}\right) \cdot \sqrt{\tilde{r}_{\mathrm{CT}}^{\mathrm{SEI}}\, \tilde{r}_{\mathrm{eon}}}}{1 + \frac{\tilde{r}_{\mathrm{CT}}^{\mathrm{SEI}}\, \tilde{r}_{\mathrm{eon}}}{\tilde{R}_{\mathrm{CT}}^{\mathrm{SEI}}\, \tilde{R}_{\mathrm{CT}}^{\mathrm{int}}} + \left(\frac{1}{\tilde{R}_{\mathrm{CT}}^{\mathrm{SEI}}} + \frac{1}{\tilde{R}_{\mathrm{CT}}^{\mathrm{int}}}\right) \cdot \sqrt{\tilde{r}_{\mathrm{CT}}^{\mathrm{SEI}}\, \tilde{r}_{\mathrm{eon}}}} \tag{S26}$$

and

$$\frac{1}{\tilde{J}_i} = \tilde{r}_i\, \tilde{d}_{\mathrm{SEI}} + \frac{\sqrt{\tilde{r}_{\mathrm{CT}}^{\mathrm{SEI}}\, \tilde{r}_{\mathrm{eon}}} + \frac{\tilde{r}_{\mathrm{CT}}^{\mathrm{SEI}}\, \tilde{r}_i^{\,2}}{\tilde{R}_{\mathrm{CT}}^{\mathrm{SEI}}\, \tilde{r}_{\mathrm{eon}}} + \frac{\tilde{r}_{\mathrm{CT}}^{\mathrm{SEI}}\, \tilde{r}_{\mathrm{eon}}}{\tilde{R}_{\mathrm{CT}}^{\mathrm{int}}}}{1 + \frac{\tilde{r}_{\mathrm{CT}}^{\mathrm{SEI}}\, \tilde{r}_{\mathrm{eon}}}{\tilde{R}_{\mathrm{CT}}^{\mathrm{SEI}}\, \tilde{R}_{\mathrm{CT}}^{\mathrm{int}}} + \left(\frac{1}{\tilde{R}_{\mathrm{CT}}^{\mathrm{SEI}}} + \frac{1}{\tilde{R}_{\mathrm{CT}}^{\mathrm{int}}}\right) \cdot \sqrt{\tilde{r}_{\mathrm{CT}}^{\mathrm{SEI}}\, \tilde{r}_{\mathrm{eon}}}}. \tag{S27}$$

With $\tilde{R}_{\mathrm{CT}}^{\mathrm{SEI}} \gg \tilde{R}_{\mathrm{CT}}^{\mathrm{int}}$, it follows:

$$\frac{1}{\tilde{j}_i} = \tilde{r}_i\,\tilde{d}_{\rm SEI} + \frac{\sqrt{\tilde{r}_{\rm CT}^{\rm SEI}\,\tilde{r}_{\rm eon}} + \frac{\tilde{r}_{\rm CT}^{\rm SEI}\,\tilde{r}_i^{\,2}}{\tilde{R}_{\rm CT}^{\rm SEI}\,\tilde{r}_{\rm eon}} + \frac{\tilde{r}_{\rm CT}^{\rm SEI}\,\tilde{r}_{\rm eon}}{\tilde{R}_{\rm CT}^{\rm int}}}{1 + \frac{\tilde{r}_{\rm CT}^{\rm SEI}\,\tilde{r}_{\rm eon}}{\tilde{R}_{\rm CT}^{\rm SEI}\,\tilde{R}_{\rm CT}^{\rm int}} + \frac{\sqrt{\tilde{r}_{\rm CT}^{\rm SEI}\,\tilde{r}_{\rm eon}}}{\tilde{R}_{\rm CT}^{\rm int}}}. \qquad \text{(S28)}$$

In the denominator of the right-hand side of (S28), $\frac{\sqrt{\tilde{r}_{\rm CT}^{\rm SEI}\,\tilde{r}_{\rm eon}}}{\tilde{R}_{\rm CT}^{\rm int}} = 10^6$ is significantly larger than $\frac{\tilde{r}_{\rm CT}^{\rm SEI}\,\tilde{r}_{\rm eon}}{\tilde{R}_{\rm CT}^{\rm SEI}\,\tilde{R}_{\rm CT}^{\rm int}} = 10^5$ and 1. Furthermore at $\tilde{k}_i = 10^4$, the term $\frac{\tilde{r}_{\rm CT}^{\rm SEI}\,\tilde{r}_{\rm eon}}{\tilde{R}_{\rm CT}^{\rm int}} = 10^{16}$ in the numerator is significantly larger than $\frac{\tilde{r}_{\rm CT}^{\rm SEI}\,\tilde{r}_i^{\,2}}{\tilde{R}_{\rm CT}^{\rm SEI}\,\tilde{r}_{\rm eon}} = 10^5$ and $\sqrt{\tilde{r}_{\rm CT}^{\rm SEI}\,\tilde{r}_{\rm eon}} = 10^{10}$. Accordingly, Eq. (S28) simplifies to:

$$\frac{1}{\tilde{j}_i} = \tilde{r}_i\,\tilde{d}_{\rm SEI} + \sqrt{\tilde{r}_{\rm CT}^{\rm SEI}\,\tilde{r}_{\rm eon}}. \qquad \text{(S29)}$$

With $\tilde{r}_i\,\tilde{d}_{\rm SEI} \ll \sqrt{\tilde{r}_{\rm CT}^{\rm SEI}\,\tilde{r}_{\rm eon}}$ we obtain finally:

$$\tilde{j}_i = \frac{1}{\sqrt{\tilde{r}_{\rm CT}^{\rm SEI}\,\tilde{r}_{\rm eon}}} = \tilde{c}_{\rm eon}^{x=0}\,\sqrt{\tilde{k}_i\,\varepsilon\,\tilde{c}_i^{\rm bulk}\,\tilde{D}_{\rm eon}}. \qquad \text{(S30)}$$

Regime (iv)

For very high rate constants around $\tilde{k}_i = 10^9$, Eq. (S25) holds and the term $2\,\tilde{r}_i\,\tilde{r}_{\rm eon}$ can be neglected since $2\,\tilde{r}_i\,\tilde{r}_{\rm eon} = 2 \cdot 10^6$ is significantly smaller than $\frac{e^{\beta}}{2}$ with $\beta \approx 10^3$. In this case, Eq. (S9) simplifies to:

$$\frac{1}{\tilde{j}_i} = \frac{\tilde{r}_i\,\tilde{r}_{\rm eon}}{\tilde{r}_i + \tilde{r}_{\rm eon}}\,\tilde{d}_{\rm SEI} + \frac{\sqrt{\tilde{r}_{\rm CT}^{\rm SEI}}}{(\tilde{r}_i + \tilde{r}_{\rm eon})^{3/2}}\;\frac{\tilde{r}_i^{\,2} + \tilde{r}_{\rm eon}^{\,2} + \left(\frac{\tilde{r}_i^{\,2}}{\tilde{R}_{\rm CT}^{\rm SEI}} + \frac{\tilde{r}_{\rm eon}^{\,2}}{\tilde{R}_{\rm CT}^{\rm int}}\right)\cdot\sqrt{\tilde{r}_{\rm CT}^{\rm SEI}\,(\tilde{r}_i + \tilde{r}_{\rm eon})}}{1 + \frac{\tilde{r}_{\rm CT}^{\rm SEI}\,\tilde{r}_{\rm eon}}{\tilde{R}_{\rm CT}^{\rm SEI}\,\tilde{R}_{\rm CT}^{\rm int}} + \left(\frac{1}{\tilde{R}_{\rm CT}^{\rm SEI}} + \frac{1}{\tilde{R}_{\rm CT}^{\rm int}}\right)\cdot\sqrt{\tilde{r}_{\rm CT}^{\rm SEI}\,(\tilde{r}_i + \tilde{r}_{\rm eon})}}. \qquad \text{(S31)}$$

With $\tilde{r}_i \ll \tilde{r}_{\rm eon}$ Eq. (S31) reduces to Eq. (S32), which can be further simplified to Eq. (S33).

$$\frac{1}{\tilde{j}_i} = \frac{\tilde{r}_i\,\tilde{r}_{\rm eon}}{\tilde{r}_i + \tilde{r}_{\rm eon}}\,\tilde{d}_{\rm SEI} + \frac{\sqrt{\tilde{r}_{\rm CT}^{\rm SEI}}}{(\tilde{r}_{\rm eon})^{3/2}}\;\frac{\tilde{r}_{\rm eon}^{\,2} + \left(\frac{\tilde{r}_i^{\,2}}{\tilde{R}_{\rm CT}^{\rm SEI}} + \frac{\tilde{r}_{\rm eon}^{\,2}}{\tilde{R}_{\rm CT}^{\rm int}}\right)\cdot\sqrt{\tilde{r}_{\rm CT}^{\rm SEI}\,\tilde{r}_{\rm eon}}}{1 + \frac{\tilde{r}_{\rm CT}^{\rm SEI}\,\tilde{r}_{\rm eon}}{\tilde{R}_{\rm CT}^{\rm SEI}\,\tilde{R}_{\rm CT}^{\rm int}} + \left(\frac{1}{\tilde{R}_{\rm CT}^{\rm SEI}} + \frac{1}{\tilde{R}_{\rm CT}^{\rm int}}\right)\cdot\sqrt{\tilde{r}_{\rm CT}^{\rm SEI}\,\tilde{r}_{\rm eon}}} \qquad \text{(S32)}$$

$$\frac{1}{\tilde{j}_i} = \frac{\tilde{r}_i\,\tilde{r}_{\mathrm{eon}}}{\tilde{r}_i + \tilde{r}_{\mathrm{eon}}}\,\tilde{d}_{\mathrm{SEI}} + \frac{\sqrt{\tilde{r}_{\mathrm{CT}}^{\mathrm{SEI}}\,\tilde{r}_{\mathrm{eon}}} + \frac{\tilde{r}_{\mathrm{CT}}^{\mathrm{SEI}}\,\tilde{r}_i^{\,2}}{\tilde{R}_{\mathrm{CT}}^{\mathrm{SEI}}\,\tilde{r}_{\mathrm{eon}}} + \frac{\tilde{r}_{\mathrm{CT}}^{\mathrm{SEI}}\,\tilde{r}_{\mathrm{eon}}}{\tilde{R}_{\mathrm{CT}}^{\mathrm{int}}}}{1 + \frac{\tilde{r}_{\mathrm{CT}}^{\mathrm{SEI}}\,\tilde{r}_{\mathrm{eon}}}{\tilde{R}_{\mathrm{CT}}^{\mathrm{SEI}}\,\tilde{R}_{\mathrm{CT}}^{\mathrm{int}}} + \left(\frac{1}{\tilde{R}_{\mathrm{CT}}^{\mathrm{SEI}}} + \frac{1}{\tilde{R}_{\mathrm{CT}}^{\mathrm{int}}}\right)\cdot\sqrt{\tilde{r}_{\mathrm{CT}}^{\mathrm{SEI}}\,\tilde{r}_{\mathrm{eon}}}}. \tag{S33}$$

With $\tilde{R}_{\mathrm{CT}}^{\mathrm{SEI}} \gg \tilde{R}_{\mathrm{CT}}^{\mathrm{int}}$, it follows:

$$\frac{1}{\tilde{j}_i} = \frac{\tilde{r}_i\,\tilde{r}_{\mathrm{eon}}}{\tilde{r}_i + \tilde{r}_{\mathrm{eon}}}\,\tilde{d}_{\mathrm{SEI}} + \frac{\sqrt{\tilde{r}_{\mathrm{CT}}^{\mathrm{SEI}}\,\tilde{r}_{\mathrm{eon}}} + \frac{\tilde{r}_{\mathrm{CT}}^{\mathrm{SEI}}\,\tilde{r}_i^{\,2}}{\tilde{R}_{\mathrm{CT}}^{\mathrm{SEI}}\,\tilde{r}_{\mathrm{eon}}} + \frac{\tilde{r}_{\mathrm{CT}}^{\mathrm{SEI}}\,\tilde{r}_{\mathrm{eon}}}{\tilde{R}_{\mathrm{CT}}^{\mathrm{int}}}}{1 + \frac{\tilde{r}_{\mathrm{CT}}^{\mathrm{SEI}}\,\tilde{r}_{\mathrm{eon}}}{\tilde{R}_{\mathrm{CT}}^{\mathrm{SEI}}\,\tilde{R}_{\mathrm{CT}}^{\mathrm{int}}} + \frac{\sqrt{\tilde{r}_{\mathrm{CT}}^{\mathrm{SEI}}\,\tilde{r}_{\mathrm{eon}}}}{\tilde{R}_{\mathrm{CT}}^{\mathrm{int}}}}. \tag{S34}$$

Since in the denominator, the term $\frac{\sqrt{\tilde{r}_{\mathrm{CT}}^{\mathrm{SEI}}\,\tilde{r}_{\mathrm{eon}}}}{\tilde{R}_{\mathrm{CT}}^{\mathrm{int}}} \approx 10^8$ is significantly smaller than $\frac{\tilde{r}_{\mathrm{CT}}^{\mathrm{SEI}}\,\tilde{r}_{\mathrm{eon}}}{\tilde{R}_{\mathrm{CT}}^{\mathrm{SEI}}\,\tilde{R}_{\mathrm{CT}}^{\mathrm{int}}} = 10^{10}$, and in the numerator, the terms $\sqrt{\tilde{r}_{\mathrm{CT}}^{\mathrm{SEI}}\,\tilde{r}_{\mathrm{eon}}} \approx 10^7$ and $\frac{\tilde{r}_{\mathrm{CT}}^{\mathrm{SEI}}\,\tilde{r}_i^{\,2}}{\tilde{R}_{\mathrm{CT}}^{\mathrm{SEI}}\,\tilde{r}_{\mathrm{eon}}} = 10^5$ are significantly smaller than $\frac{\tilde{r}_{\mathrm{CT}}^{\mathrm{SEI}}\,\tilde{r}_{\mathrm{eon}}}{\tilde{R}_{\mathrm{CT}}^{\mathrm{int}}} = 10^{16}$, Eq. (S34) simplifies to:

$$\frac{1}{\tilde{j}_i} = \frac{\tilde{r}_i\,\tilde{r}_{\mathrm{eon}}}{\tilde{r}_i + \tilde{r}_{\mathrm{eon}}}\,\tilde{d}_{\mathrm{SEI}} + \frac{\frac{\tilde{r}_{\mathrm{CT}}^{\mathrm{SEI}}\,\tilde{r}_{\mathrm{eon}}}{\tilde{R}_{\mathrm{CT}}^{\mathrm{int}}}}{\frac{\tilde{r}_{\mathrm{CT}}^{\mathrm{SEI}}\,\tilde{r}_{\mathrm{eon}}}{\tilde{R}_{\mathrm{CT}}^{\mathrm{SEI}}\,\tilde{R}_{\mathrm{CT}}^{\mathrm{int}}}} = \frac{\tilde{r}_i\,\tilde{r}_{\mathrm{eon}}}{\tilde{r}_i + \tilde{r}_{\mathrm{eon}}}\,\tilde{d}_{\mathrm{SEI}} + \tilde{R}_{\mathrm{CT}}^{\mathrm{SEI}}. \tag{S35}$$

Furthermore, since $\tilde{R}_{\mathrm{CT}}^{\mathrm{SEI}} \ll \frac{\tilde{r}_i\,\tilde{r}_{\mathrm{eon}}}{\tilde{r}_i + \tilde{r}_{\mathrm{eon}}}\,\tilde{d}_{\mathrm{SEI}}$, we obtain finally:

$$\frac{1}{\tilde{j}_i} = \frac{\tilde{r}_i\,\tilde{r}_{\mathrm{eon}}}{\tilde{r}_i + \tilde{r}_{\mathrm{eon}}}\,\tilde{d}_{\mathrm{SEI}}. \tag{S36}$$

and

$$\tilde{j}_i = \frac{\widetilde{D}_i\,\varepsilon\,\tilde{c}_i^{\mathrm{bulk}} + \widetilde{D}_{\mathrm{eon}}\,\tilde{c}_{\mathrm{eon}}^{x=0}}{\tilde{d}_{\mathrm{SEI}}}. \tag{S37}$$

6. Physical Meaning of the Nernst-Einstein Equation for Electron Diffusion

A general description of electron transport is given by the Onsager relation for the electron flux:

$$J_{\mathrm{eon}} = -\frac{\sigma_{\mathrm{eon}}}{\mathrm{F}^2} \cdot \frac{d\tilde{\mu}_{\mathrm{eon}}}{dx} \tag{S38}$$

with σ_{eon} and $\tilde{\mu}_{\mathrm{eon}}$ denoting the Onsager transport coefficient of the electrons and the electrochemical potential of the electrons, respectively. Since there is no electric potential gradient in the SEI, $\tilde{\mu}_{\mathrm{eon}}$ can be replaced by the chemical potential of the electrons, μ_{eon}.

At low electron concentrations, the activity coefficient of the electrons can be set to unity, resulting in the following relation for the chemical potential gradient of the electrons:

$$\frac{d\mu_{\mathrm{eon}}}{dx} = \mathrm{R}T\frac{d\ln c_{\mathrm{eon}}}{dx}. \tag{S39}$$

The diffusion coefficient and the Onsager transport coefficient of the electrons can be written as:[8]

$$D_{\mathrm{eon}} = \lim_{t\to\infty}\frac{d}{dt}\left[\frac{1}{6N_{\mathrm{eon}}}\left(\sum_{i=1}^{N_{\mathrm{eon}}}\left(\Delta\vec{R}_i(t)\right)^2\right)\right] \tag{S40}$$

$$\sigma_{\mathrm{eon}} = \lim_{t\to\infty}\frac{d}{dt}\left[\frac{e^2}{6V\,\mathrm{k_B}T}\left(\sum_{i=1}^{N_{\mathrm{eon}}}\Delta\vec{R}_i(t)\right)^2\right] \tag{S41}$$

with $\Delta\vec{R}_i(t)$ denoting the displacement vector of electron *i.*

At low electron concentrations, correlated movements of different electrons can be neglected, implying that $\left(\sum_{i=1}^{N_{\mathrm{eon}}}\left(\Delta\vec{R}_i(t)\right)^2\right) = \left(\sum_{i=1}^{N_{\mathrm{eon}}}\Delta\vec{R}_i(t)\right)^2$. This results in a Nernst-Einstein equation for electron transport:

$$\sigma_{\mathrm{eon}} = \frac{N_{\mathrm{eon}}e^2D_{\mathrm{eon}}}{V\,\mathrm{R}T} = \frac{c_{\mathrm{eon}}\mathrm{F}^2D_{\mathrm{eon}}}{\mathrm{R}T}. \tag{S42}$$

Inserting $\frac{d\mu_{\mathrm{eon}}}{dx}$ = RT $\frac{d\ln c_{\mathrm{eon}}}{dx}$ and the Nernst-Einstein equation into the Onsager relation for the flux results in Fick's first law for electron diffusion:

$$J_{\mathrm{eon}} = -D_{\mathrm{eon}} \cdot \frac{dc_{\mathrm{eon}}}{dx}. \tag{S43}$$

References

[1] A. H. Scott, H. L. Curtis, *J. Res. Natl. Bur. Stand.* **1939**, *22*, 747–775.

[2] A. Kakimoto, *Rev. Sci. Instrum.* **1972**, *43*, 763–765, DOI: 10.1063/1.1685753.

[3] M. A. Rivas, S. M. Pereira, N. Banerji, T. P. Iglesias, *J. Chem. Thermodynamics* **2004**, *36*, 183–191. DOI: 10.1016/j.jct.2003.11.007.

[4] S. Kranz, T. Kranz, T. Graubner, Y. Yusim, L. Hellweg, B. Roling, *Batteries Supercaps* **2019**, *2*, 1026–1036. DOI: 10.1002/batt.201900110.

[5] T. Kranz, S. Kranz, V. Miß, J. Schepp, B. Roling, *J. Electrochem. Soc.* **2017**, *164*, A3777–A3784. DOI: 10.1149/2.1171714jes.

[6] F. T. Krauss, I. Pantenburg, V. Lehmann, M. Stich, J. O. Weiershäuser, A. Bund, B. Roling, *J. Am. Chem. Soc.* **2024**, *146*. 19009–19018, DOI: 10.1021/jacs.4c03029.

[7] Z. Siroma, N. Fujiwara, S. Yamazaki, M. Asahi, T. Nagai, T. Ioroi, *Electrochim. Acta* **2015**, *160*, 313–322. DOI: 10.1016/j.electacta.2015.02.065.

[8] N. M. Vargas-Barbosa, B. Roling, *ChemElectroChem* **2020**, *7*, 367–385. DOI: 10.1002/celc.201901627.
